# Polyphosphatases have a polyphosphate-independent influence on the virulence of *Cryptococcus neoformans*

**DOI:** 10.1128/iai.00072-25

**Published:** 2025-03-12

**Authors:** Kabir Bhalla, Eddy Sánchez León-Hing, Yu-Hsuan Huang, Victoria French, Guanggan Hu, Jen Wang, Matthias Kretschmer, Xianya Qu, Raphaell Moreira, E. Johan Foster, Pauline Johnson, James W. Kronstad

**Affiliations:** 1 Michael Smith Laboratories, University of British Columbia 98687 , Vancouver, British Columbia, Canada; 2 Department of Microbiology and Immunology, University of British Columbia 198130 , Vancouver, British Columbia, Canada; 3 Department of Chemical and Biological Engineering, BioProducts Institute, University of British Columbia 536638 , Vancouver, British Columbia, Canada; NIH, NIAID, Washington, DC, USA

**Keywords:** fungal pathogenesis, immunity, capsule, melanin, mitochondrial metabolism, polyphosphate

## Abstract

*Cryptococcus neoformans,* an invasive basidiomycete fungal pathogen, causes one of the most prevalent, life-threatening diseases in immunocompromised individuals and accounts for ~19% of AIDS-associated deaths. Therefore, understanding the pathogenesis of *C. neoformans* and its interactions with the host immune system is critical for developing therapeutics against cryptococcosis. Previous studies demonstrated that *C. neoformans* cells lacking polyphosphate (polyP), an immunomodulatory polyanionic storage molecule, display altered cell surface architecture but unimpaired virulence in a murine model of cryptococcosis. However, the relevance of cell surface changes and the role of hyperaccumulation of polyP in the virulence of *C. neoformans* remain unclear. Here we show that mutants with abundant polyP due to loss of the polyphosphatases Xpp1 and Epp1 are attenuated for virulence. The double mutant differed from the wild type during disease by demonstrating a higher fungal burden in disseminated organs at the experimental endpoint and by provoking an altered immune response. An analysis of triple mutants lacking the polyphosphatases and the Vtc4 protein for polyP synthesis also caused attenuated virulence in mice, thus suggesting an influence of Xpp1 and/or Epp1 independent of polyP levels. A more detailed characterization revealed that Xpp1 and Epp1 play multiple roles by contributing to the organization of the cell surface, virulence factor production, the response to stress, and mitochondrial function. Overall, we conclude that polyphosphatases have additional functions in the pathobiology of *C. neoformans* beyond an influence on polyP levels.

**IMPORTANCE:**

Cryptococcus neoformans causes one of the most prevalent fungal diseases in people with compromised immune systems and accounts for ~19% of AIDS-associated deaths worldwide. The continual increase in the incidence of fungal infections and limited treatment options necessitate the development of new antifungal drugs and improved diagnostics. Polyphosphate (polyP), an under-explored biopolymer, functions as a storage molecule, modulates the host immune response, and contributes to the ability of some fungal and bacterial pathogens to cause disease. However, the role of polyP in cryptococcal disease remains unclear. In this study, we report that the polyphosphatase enzymes that regulate polyP synthesis and turnover contribute to the virulence of *C. neoformans* in a mouse model of cryptococcosis. The polyphosphatases influenced the survival of *C. neoformans* in macrophages and altered the host immune response. In addition, the mutants lacking the enzymes have changes in cell surface architecture and size, as well as defects in both mitochondrial function and the stress response. By using mutants defective in the polyphosphatases and polyP synthesis, we demonstrate that many of the phenotypic contributions of the polyphosphatases are independent of polyP.

## INTRODUCTION

Fungi are underappreciated and neglected as major pathogens of humans despite over a billion individuals being impacted each year worldwide (1–4). As an example, cryptococcal disease is responsible for ~300,000 cases of meningoencephalitis each year and is the second leading cause of AIDS-associated deaths in sub-Saharan Africa (1–4). Acquired by inhalation of basidiospores or desiccated yeast cells, *C. neoformans* has a propensity to disseminate to the central nervous system and cause life-threatening disease in immunocompromised individuals (2). Currently, the treatment for cryptococcosis involves long-term therapy, but limited antifungal agents are available (5, 6). The continual increase in the incidence of fungal infections and the notoriously difficult nature of treatment due to host toxicity and anti-cryptococcal drug resistance highlights the critical need to develop new antifungal drugs and improved diagnostics (2, 5, 6). Cryptococcal disease is also a more general concern for public health because *Cryptococcus gattii*, a closely related sibling species with shared virulence factors, can cause cryptococcosis in immunocompetent individuals without any underlying health conditions (2, 4).

As a facultative intracellular pathogen, *C. neoformans* employs several host damage mechanisms and virulence factors that help it evade host immunity, persist in the intracellular environment, and resist the unfavorable conditions of the phagolysosome (7–16). One of the major virulence factors is the polysaccharide capsule which modulates immune responses and prevents recognition and phagocytosis by masking immunoreactive pathogen-associated molecular patterns (PAMPs) such as chitin and chitosan (11). Glucuronoxylomannan (GXM) and glucuronoxylomannogalactan (GXMGal), the two major components of the capsule, have additional functions in the depletion of complement and inhibition of neutrophil migration (11). Moreover, the capsule is involved in protecting the fungus from oxidative stresses (11). In addition, *C. neoformans* produces melanin, an immunogenic natural pigment and antioxidant, which further protects against oxidative damage and fluctuations in temperature (12). Production of melanin also confers resistance to antifungal drugs such as amphotericin B and contributes to fungal dissemination to the brain (12, 13). Lastly, titanization, or formation of morphologically giant cells characterized by thicker cell walls and large vacuoles, is a unique virulence factor that allows *C. neoformans* to evade phagocytosis (14, 15).

Upon inhalation, *C. neoformans* cells colonize lung tissue and encounter resident immune cells (17, 18). β-glucans and chitin, as well as chitosan present on the cell wall, act as fungal PAMPs and are recognized by immune cells through pattern recognition receptors (PRRs) such as Toll-like receptors (TLRs) and C-type lectin receptors (CLRs) to trigger appropriate cytokine and phagocytic responses (19, 20). Alveolar macrophages are the first line of defense to detect and phagocytose cryptococcal cells, with subsequent recruitment of monocytes and dendritic cells (DCs) to the lung (17–21). These recruited DCs also internalize yeast cells and present antigens to naïve CD4+ T cells in the lymph nodes (17, 21). As with other intracellular pathogens, the polarization and induction of IFN-γ-, IL-6-, and IL-17-secreting T helper 1 (Th1) and T helper 17 (Th17) cells promote pathogen clearance (21). In fact, mice lacking IFN-γ receptors display high susceptibility to cryptococcosis and have increased fungal burdens (22). Furthermore, mice deficient in both Th1 and Th17 responses display heightened mortality to cryptococcal infections compared with mice deficient in only Th1 responses (23). The commonly studied wild-type *C. neoformans* strain, H99, is known to induce a non-protective Th2 immune response that involves the production of IL-4 in BALB/c mice (7). Th2 responses are maladaptive and are commonly associated with promoting fungal dissemination and proliferation (24).

Polyphosphate (polyP), an under-explored, polyanionic inorganic biopolymer, serves as a phosphorus and energy storage molecule (25–30). In fungi, polyP is found in different cellular compartments where it complexes with various inorganic and organic cations (26). Recent findings also identified the involvement of this polyanion in osmoregulation, stress adaptation, cell membrane formation, biofilm development, quorum sensing, and protein targeting (27–30). PolyP is also known to contribute to virulence in many bacterial pathogens including *Salmonella* and *Mycobacterium* (26). More specifically, prokaryotic polyP inhibits the polarization of M1 macrophages, suppresses inducible nitric oxide synthase (iNOS), and impedes major histocompatibility complex class II (MHC II) activation in the host (26). PolyP also plays a myriad of roles in fungi. For instance, in both *C. neoformans* and *Candida albicans*, polyP is involved in cation resistance and detoxification (31, 32). Emerging research on cell cycle progression in *Saccharomyces cerevisiae* also revealed a new role for polyP in DNA replication and dNTP synthesis (33). In *Ustilago maydis*, another basidiomycetous fungal pathogen, polyP directly influences virulence and symptom development (25, 34).

A number of key proteins are involved in polyP metabolism (35–37). In *S. cerevisiae* (and other fungi), synthesis and vacuolar translocation of polyP is mediated by a polyP synthetase known as vacuolar transporter chaperone 4 or Vtc4 (36). Cells deficient in the Vtc4 polyP synthetase have no detectable polyP, and for *C. neoformans,* this results in altered proliferation in the host and an impaired ability to trigger blood coagulation (32). In addition, the genes *XPP1* and *EPP1* in *C. neoformans* encode putative exo- and endopolyphosphatases that catalyze the hydrolysis or mobilization of inorganic polyP in response to phosphate-limiting conditions (32). Cells lacking exo- and endopolyphosphatases have significantly increased levels of polyP (32). In this study, we demonstrate that mutants lacking Xpp1 and Epp1 display attenuated virulence in mice, altered cell surface architecture and virulence factor elaboration, and defects in mitochondrial function. Importantly, an analysis of a triple mutant lacking Xpp1, Epp1, and Vtc4 revealed that the polyphosphatases contribute to virulence by mechanisms independent of polyP.

## RESULTS

### Mutants lacking polyphosphatases exhibit attenuated virulence

**Fig 1:**
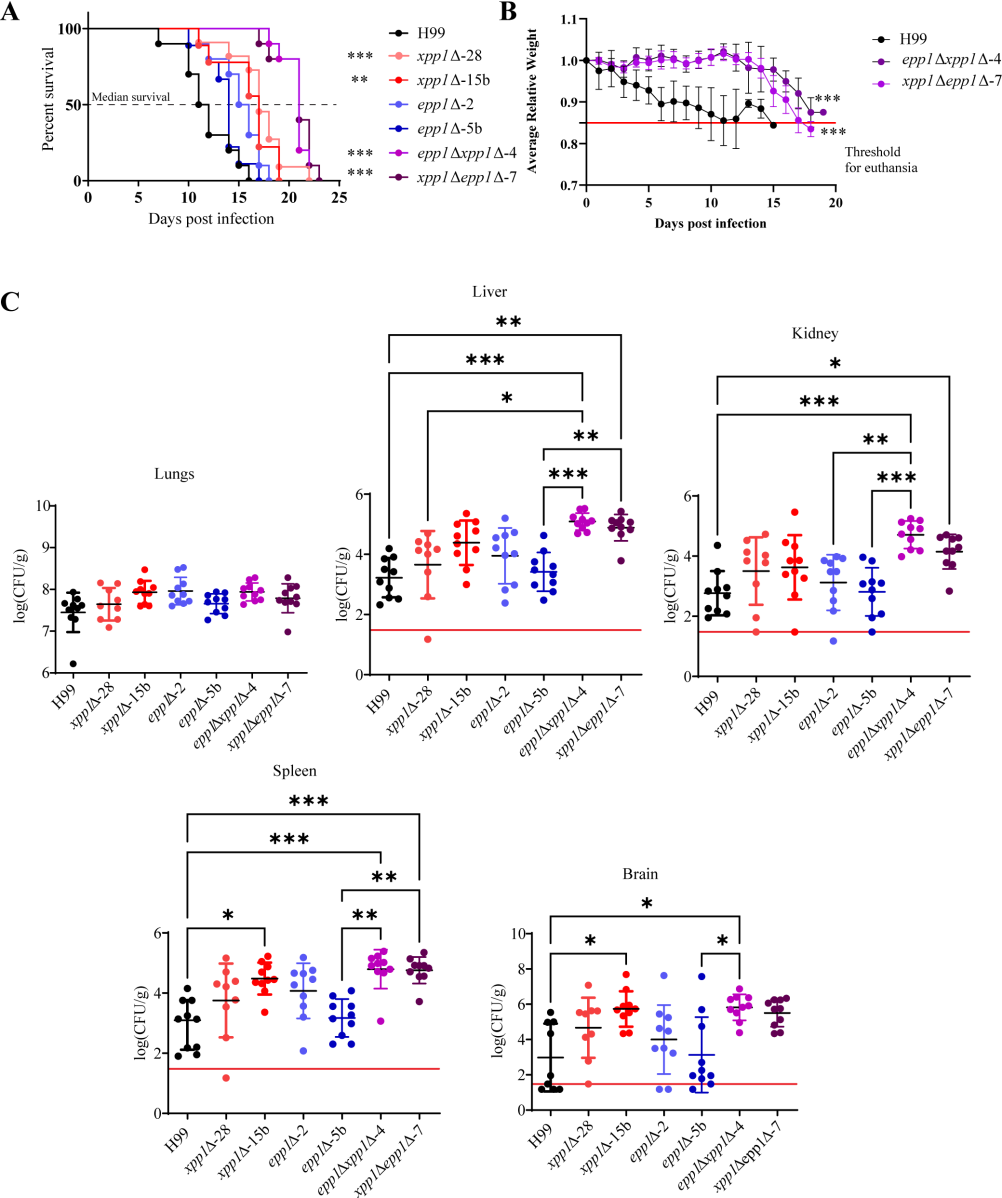
Loss of polyphosphatases influences virulence in a mouse model of cryptococcosis. (**A**) Survival of BALB/c mice intranasally inoculated with cells of WT strain (H99), two independent *xpp1*∆ mutants, two *epp1*∆ mutants, or two *xpp1*∆*epp1*∆ mutants. The black dashed horizontal line indicates the median survival of mice (**B**) The average daily mouse weight, relative to initial weight, was used to track disease progression during the first 20 days post-infection (dpi). A solid red horizontal line indicates the threshold for euthanasia at 85% of the initial weight. Error bars represent the standard deviation (SD) from all surviving mice (*n* = 10). (**C**) CFUs were measured to determine fungal burden in the lungs, liver, kidney, spleen, and brain for mice infected with each strain. The solid red horizontal line indicates the limit of detection for each organ. Data are presented as mean ± SD. Significance indicated as *P < 0.05; **P < 0.01; ***P < 0.001; one-way ANOVA or log-rank test.

Previously, we demonstrated that the impaired synthesis of polyP in a *vtc4*Δ mutant does not influence virulence in a mouse inhalational model of cryptococcosis (32). However, the influence of enhanced polyP levels and polyphosphatases on the virulence of *C. neoformans* has not been evaluated. Given that defects in polyphosphatases reduce virulence in bacterial and other fungal pathogens (38, 39), we hypothesized that overaccumulation of intracellular polyP would impact the virulence of *C. neoformans*. To test this idea, we assessed the ability of *xpp1*∆, *epp1*∆, and *xpp1*∆*epp1*∆ mutants (two independent strains of each) with hyperaccumulation of polyP to cause disease in a mouse model of cryptococcosis. We challenged BALB/c mice by intranasal inoculation with the wild-type (WT) strain (H99) or the mutant strains (*xpp1*∆, *epp1*∆, or *xpp1*∆*epp1*∆), and monitored disease progression and survival. In contrast to the WT strain, which caused the infected mice to succumb to the disease by day 16, the virulence of the mutants was attenuated such that the infected mice exhibited delayed disease symptoms and mortality (Fig. 1A). More specifically, we found that disease caused by both independent *xpp1*∆ single deletion mutants (but not the *epp1*∆ single deletion mutants) was significantly attenuated compared to infection with the WT strain (Fig. 1A). Virulence was further attenuated upon infection with the two independent *xpp1*∆ *epp1*∆ double deletion mutants. The difference in virulence for the double mutants was also evident when disease progression was monitored by weight loss for 20 days post-inoculation. Mice infected with the WT strain lost weight for 15 days, whereas the mice infected with the double mutant showed a more gradual weight decline prior to euthanasia (Fig. 1B). An examination of fungal burden in organs harvested from infected mice at the experimental endpoint revealed comparable levels for the mutant and WT strains in the lungs, but increased colony-forming units (CFUs) in the liver, kidney, spleen, and brain of mice infected with the double mutants (Fig. 1C). Mice infected with the *xpp1*∆*epp1*∆ mutants also exhibited disrupted lung architecture at endpoint (Fig. S1). More specifically, the lungs of double mutant infected mice showed more severe lung pathology, characterized by fungi growing in all regions of the lungs and displacing lung tissue (formation of cryptococcomas), compared to the lungs of WT-infected mice. Consistent with lung damage, we noted that the mice infected with the mutant showed labored breathing at the later stage of the disease. Overall, these experiments revealed a contribution of Xpp1 and Epp1 to virulence and the accumulation of fungal cells in organs beyond the lung.

### Mice infected with *xpp1*∆ *epp1*∆ mutants show reduced fungal burdens and an altered immune response early in the disease

**Fig 2:**
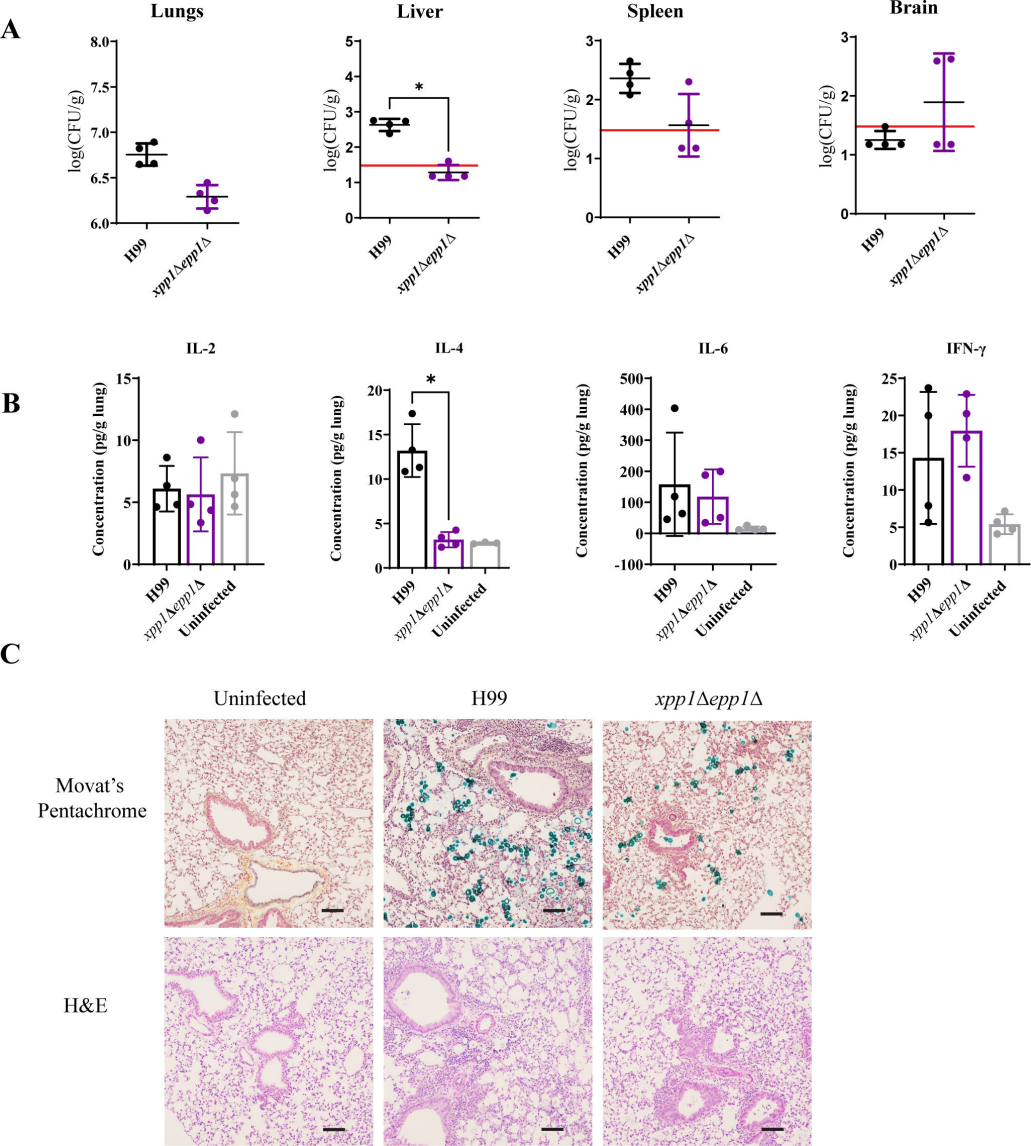
The *xpp1*∆*epp1*∆ mutant provokes an altered immune response at day 7 and attenuates virulence in the mouse model. (**A**) CFUs were measured to determine fungal burden in lungs, liver, spleen, and brain for mice infected with WT, or *xpp1*∆*epp1*∆, or treated with physiological saline (uninfected) at 7 dpi. The solid red horizontal line indicates the limit of detection for each organ. (**B**) Lung cytokine profiles of BALB/c mice infected with indicated strains or treated with physiological saline at 7 days post-infection. Cytokines were extracted from lung tissue using a mixer mill and quantified using a cytometric bead array (CBA) mouse inflammation kit. Data are presented as mean ± SD, (*n* = 4) and are representative of two independent experiments. Significance indicated as *P < 0.05; one-way ANOVA with Tukey’s test. (**C**) Representative histopathological micrographs of lung tissue from mice infected with WT (H99) or *xpp1*∆*epp1*∆ mutants, collected at 7 dpi and stained with H&E or Movat’s Pentachrome. Scale bar 100 µm.

Given the delayed disease symptoms and mortality of the *xpp1*∆ *epp1*∆-infected mice during our analysis of survival, we next tested whether mutants lacking the polyP degrading enzymes were impaired in establishing infections early in the host. To address this question, we monitored disease and measured fungal load at 7 days post-inoculation. We found that mice infected with the *xpp1*Δ *epp1*Δ mutant showed fungal burdens in the brain, lungs, and spleen that were comparable to those of the WT strain (Fig. 2A). By contrast, the fungal load in the liver was significantly reduced for the mice infected with the double mutant compared to the WT strain.

We examined the disease progression more closely by measuring lung cytokine profiles and found a significantly reduced level of IL-4 in mice infected with the double mutant compared to the WT strain (Fig. 2B). Given the influence of IL-4 on T-cell differentiation and macrophage activation, this result suggests that a loss of the polyphosphatases altered the perception of fungal cells by the immune system to result in a skewed response. A further histopathology analysis on day 7 time revealed pathological differences between the mice infected with the WT strain versus the *xpp1*∆ *epp1*∆ double mutant that was consistent with our fungal burden analyses (Fig. 2C). Specifically, we observed that the lungs of WT-infected mice exhibited a more extensive and widespread presence of fungal cells (stained dark bluish green by Movat’s Pentachrome) around the bronchioles. By contrast, fungal cells in the *xpp1*∆ *epp1*∆ infected mice were less frequently observed. In addition, the lungs of WT-infected mice showed greater infiltration of host immune cells surrounding the airways and blood vessels (Fig. 2C). This observation prompted a flow cytometry examination of immune cell populations in the lungs of infected mice and mice treated with physiological saline as a control at day 7. We found that the lungs of the BALB/c mice infected with the double mutant had significantly reduced numbers of CD45+ cells and diminished lymphoid populations compared with those infected with the WT strain (Fig. S2 to S4). In particular, there were decreased numbers of both CD4+ and CD8+ T cells, but no differences in B cell or Ly6C^-^ macrophage numbers were observed (Fig. S2 to S4). There was also a significant reduction in the number of innate immune cells such as neutrophils and eosinophils (Fig. S4). The mice infected with the double mutant also exhibited decreased numbers of monocyte-derived macrophages but not monocytes (Fig. S4). Mice infected with the *xpp1*∆ *epp1*∆ mutant also had diminished type 2 conventional dendritic cells (cDC2) compared to WT-infected mice in the lung (Fig. S3C). These results suggest that the mutants deficient in the polyphosphatases stimulate a weaker immune response compared to infection with the WT strain which, in turn, might delay or prevent clearance of the double mutant from infected tissue. Taken together, these findings suggested that the polyphosphatases, and perhaps a balance of polyP levels, were required for *C. neoformans* to adapt to the host environment and initiate disease. Our observations led us to hypothesize that the *xpp1*∆ *epp1*∆ mutants initially struggle to establish infection and may stimulate weak and altered immune responses in the BALB/c mice early in infection, thereby allowing the mutants to persist in the lung (and other organs) at sufficient levels to eventually cause disease.

### Mutants defective in both polyP synthesis and polyphosphatases are attenuated for virulence

**Fig 3:**
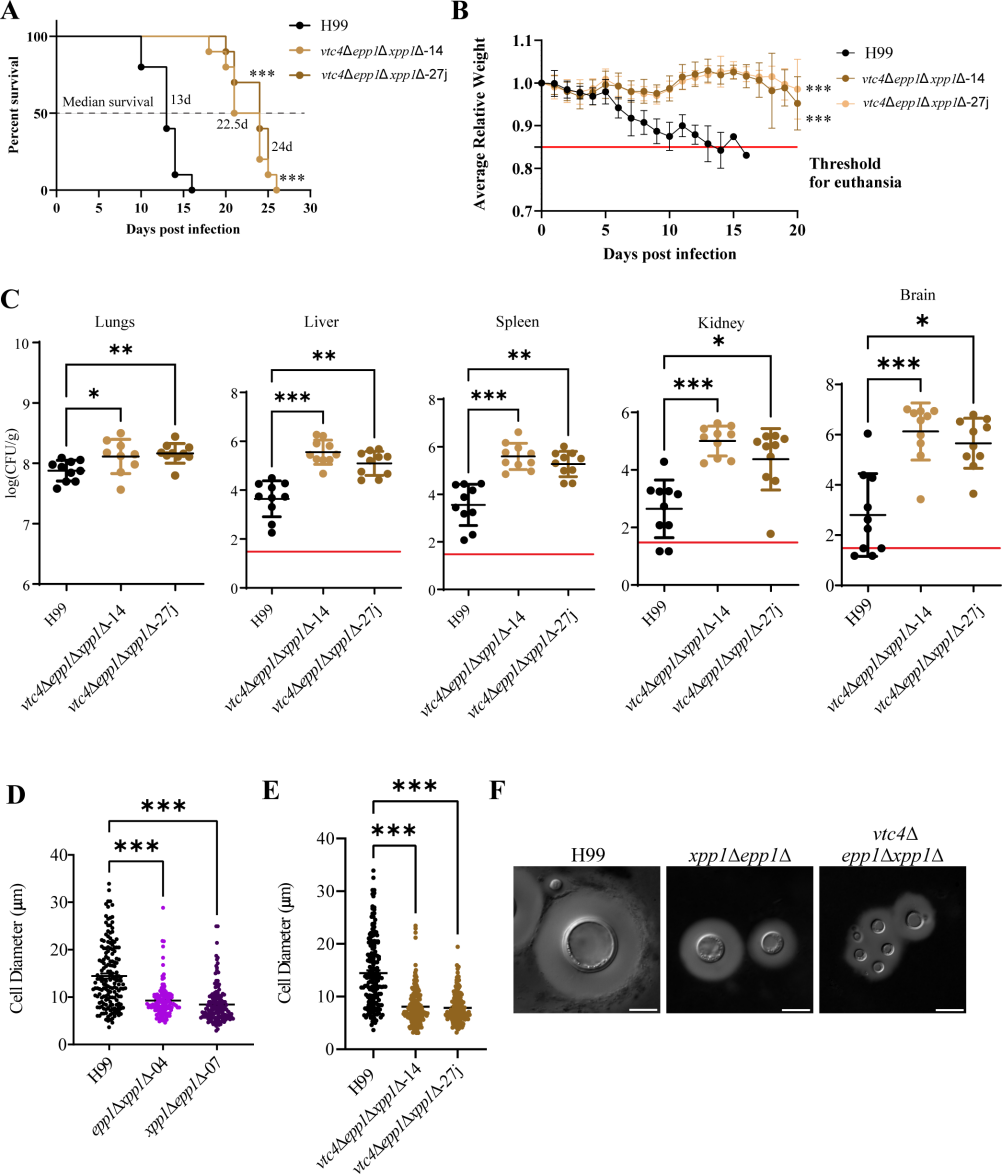
Loss of polyP synthesis and degradation enzymes impacts virulence in a mouse model of cryptococcosis. (**A**) Survival of BALB/c mice intranasally inoculated with cells of the WT strain or two independent *vtc4*∆*epp1*∆*xpp1*∆ (∆∆∆) mutants. (**B**) The average daily mouse weight, relative to initial weight, was used to track disease progression during the first 20 days post-infection. A solid red horizontal line indicates the threshold for euthanasia at 85% of the initial weight. Error bars represent standard error of means (SEM) from all surviving mice (*n* = 10). (**C**) CFUs were measured to determine fungal burden in the lungs, liver, kidney, spleen, and brain for mice infected with each strain. The solid red horizontal line indicates the limit of detection for each organ. Data are presented as mean ± SD. Significance indicated as *P < 0.05; **P < 0.01; ***P < 0.001; one-way ANOVA or log-rank test. (**D and E**) Yeast cells of the WT strain, two independent *xpp1*∆*epp1*∆ mutants, or two *vtc4*∆*epp1*∆*xpp1*∆ (∆∆∆) mutants were isolated from the lungs of infected mice (*n* = 10) and (**F**) stained with India ink to visualize polysaccharide capsules. Cell size was measured manually and at least 100 cells were analyzed per strain. Representative differential interference contrast (DIC) micrographs are shown. Scale bar 10 µm.

We previously demonstrated that the *xpp1*∆*epp1*∆ double mutant hyperaccumulates polyP (32). To determine whether this accumulation contributed to the attenuated virulence and altered immune response for the double mutant, we constructed two independent triple deletion mutants lacking the *vtc4*, *xpp1*Δ, and *epp1*Δ genes (designated *vtc4*Δ*epp1*Δ*xpp1-14 and vtc4*Δ*epp1*Δ*xpp1*-27j). Similar to the *vtc4*∆ single mutant, the triple deletion mutants exhibited impaired polyP accumulation (characterized by the absence of detectable polyP) and enhanced susceptibility to zinc (Fig. S5). We challenged mice by intranasal inoculation with the WT strain or the two independent triple deletion mutants and monitored disease progression. In contrast to the WT strain, which caused all infected mice to succumb to the disease by day 16, each of the triple mutants was attenuated for virulence, and the mice exhibited delayed disease symptoms and mortality, with the last infected mouse euthanized at day 26 (Fig. 3A). Mice infected with the two independent triple mutants had a median survival of 22.5 days and 24 days, respectively, compared to mice infected with the WT strain, which had a median survival of only 13 days. The observed impact on virulence was quite similar to the results for mice infected with the double mutants, including the fungal burden and tissue damage to the lung observed by histopathology (Fig. 1; Fig. S1). Similar to mice infected with the *xpp1*Δ*epp1*Δ mutants, mice infected with the triple mutants also showed severe lung pathology and displacement of lung tissue, and we noted that the lungs of mice infected with mutants impaired in polyP mobilization were enlarged and difficult to homogenize. We also observed accumulation of inflammatory infiltrates at the margin of infected areas in the triple mutant-infected lungs, suggesting uncontrolled growth of this mutant in the lung tissue (Fig. S1). The mice infected with the *vtc4*∆ *epp1*∆ *xpp1*∆ mutants also showed a more gradual decline in weight loss compared to mice infected with the WT strain (Fig. 3B). Moreover, mice infected with the triple mutants exhibited significantly increased fungal burdens in the lungs, livers, kidneys, spleens, and brains at the experimental endpoint (Fig. 3C). We confirmed the similarity in attenuation of virulence for the double and triple mutants in an additional survival assay (Fig. S6).

We were intrigued by the observed elevated fungal burden for the double and triple mutants in the infected organs. Given that phosphate acts as a signal for small cell induction and that decreased cell size is conducive for extrapulmonary dissemination, we hypothesized that the elevated burden phenotype observed in mice infected with the double and triple deletion mutants was due to smaller yeast cell size *in vivo* (40). Indeed, our analysis of *C. neoformans* cells isolated from lung homogenates revealed that cells of the double mutant defective in the polyphosphatases and cells of the triple mutant were significantly smaller in diameter compared to WT cells *in vivo* (Fig. 3D through F). Taken together, these experiments indicated that the attenuated virulence of the double mutant was not due to hyperaccumulation of polyP and suggested that the polyphosphatases make other contributions to virulence (and cell size) independent of polyP.

### Mutants lacking the polyphosphatases are impaired for uptake and survival in macrophages

**Fig 4:**
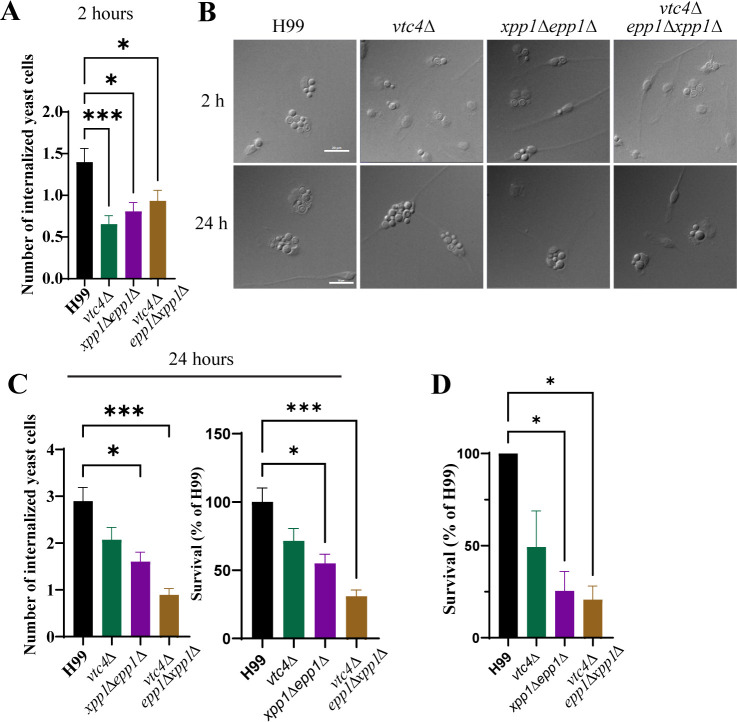
Defects in polyP metabolism reduce uptake and survival in murine macrophages. (**A**) Uptake of opsonized WT, *vtc4*∆, *xpp1*∆*epp1*∆, or *vtc4*∆*epp1*∆*xpp1*∆ (∆∆∆) strains in bone-marrow-derived macrophages are shown after 2 hours of interaction by counting the number of internalized yeast cells per macrophage. (**B**) Representative DIC micrographs of bone-marrow-derived primary macrophages (BMDMs) with internalized yeast cells at 2 hours and 24 hours of interaction are shown. Scale bar 30 µm. (**C**) Analysis of accumulation and survival of opsonized WT, *vtc4*∆, *xpp1*∆*epp1*∆, or *vtc4*∆*epp1*∆*xpp1*∆ (∆∆∆) strain in bone-marrow-derived macrophages is shown after 24 hours of interaction by counting the number of internalized yeast cells per macrophage. (**D**) Survival of opsonized WT, *vtc4*∆, *xpp1*∆*epp1*∆, or *vtc4*∆*epp1*∆*xpp1*∆ in bone-marrow-derived macrophages after 24 hours of interaction was measured by counting colony-forming units and presented as a percentage of H99 survival. Data are presented as mean ± SEM and representative of at least three independent experiments. Significance indicated as *P < 0.05; **P < 0.01; ***P < 0.001; Kruskal-Wallis nonparametric test.

To investigate other contributions of the polyphosphatases apart from polyP accumulation that might account for the attenuated virulence of the double and triple mutants, we characterized additional phenotypes relevant to proliferation in the host. We first examined the interactions of the mutants with murine macrophages by assessing the uptake and survival of opsonized yeast cells. We found that the mutants showed reduced uptake by bone-marrow-derived primary macrophages (BMDMs) (Fig. 4A and B). That is, the number of internalized yeast cells 2 hours post-infection was markedly reduced for the *vtc4*∆, *xpp1*∆ *epp1*∆, and *vtc4*∆ *epp1*∆ *xpp1*∆ mutant strains compared to the WT strain. However, only the *xpp1*∆ *epp1*∆ and *vtc4*∆ *epp1*∆ *xpp1*∆ mutants showed significantly lower accumulation at 24 hours post-infection suggesting reduced survival in the primary macrophages (Fig. 4B and C). In other words, the number of internalized yeast cells was significantly reduced at 24 hours for mutants lacking polyphosphatases, but not for the *vtc4*∆ mutant. We confirmed the reduced survival for the double and triple mutants by counting CFUs after macrophage lysis (Fig. 4D). We had previously found that the *vtc4*∆ mutant behaved like WT in terms of uptake and survival in the J774A.1 murine macrophage-like cell line (32). The difference with our current observation of reduced uptake for the *vtc4*∆ mutant may be due to the use of primary phagocytes versus the cell line and the conditions for opsonization. Taken together, our observations on phagocytosis suggest that loss of the polyphosphatases impacts uptake by and survival within macrophages.

### Loss of Xpp1 and Epp1 alters cell surface architecture and influences the elaboration of virulence factors

**Fig 5:**
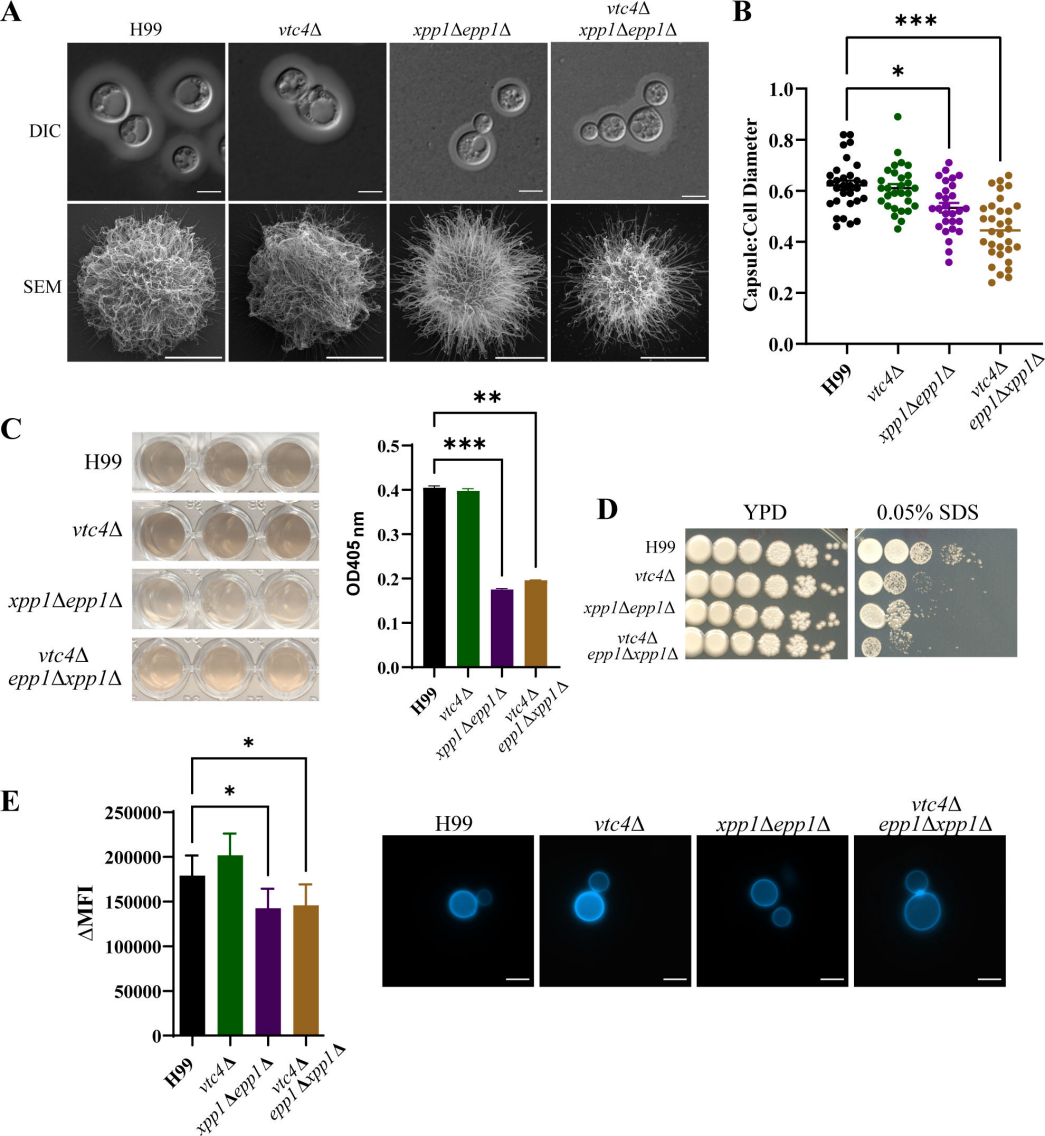
Mutants deficient in polyP mobilization have altered cell surface architecture. (**A**) WT, *vtc4*∆, *xpp1*∆*epp1*∆, or *vtc4*∆*epp1*∆*xpp1*∆ strains were inoculated into capsule-inducing medium (CIM) and incubated at 30℃ for 48 hours. Capsule formation and structure were assessed by India ink staining or SEM for the indicated strains. (**B**) The ratio of capsule thickness to cell diameter was quantified for at least 50 cells per strain. (**C**) WT, *vtc4*∆, *xpp1*∆*epp1*∆, or *vtc4*∆*epp1*∆*xpp1*∆ strains were inoculated into L-DOPA liquid medium and incubated at 30℃ for 48 hours. Melanin production was quantified using OD_405_ values. (**D**) Indicated strains were serially diluted and spotted onto solid YPD agar with or without 0.05% SDS. The plates were then incubated at 30°C for 5 days before being photographed. (**E**) WT, *vtc4*∆, *xpp1*∆*epp1*∆, or *vtc4*∆*epp1*∆*xpp1*∆ strains were stained with calcofluor white (CFW, 100 µg/mL) to visualize chitin. Chitin content was quantified using flow cytometry and analyzed by FlowJo. ΔMFI = change in mean fluorescence intensity. Scale bar 5 µm. Data are presented as mean ± SEM and representative of at least three independent experiments. Significance indicated as *P < 0.05; **P < 0.01; ***P < 0.001; one-way ANOVA.

The attenuated virulence of the *xpp1*∆*epp1*∆ and *vtc4*∆*epp1*∆*xpp1*∆ mutants in the murine inhalational model and reduced survival in murine macrophages suggested that the Xpp1 and Epp1 polyphosphatases could potentially influence major virulence factors. For example, the polyphosphatase mutants might exhibit differences in capsular architecture. To test this idea, we investigated the cell surface by India ink staining and scanning electron microscopy (SEM) and found cell surface architectural changes for the mutants. In particular, the *xpp1*∆ *epp1*∆ mutant was defective in the elaboration of the polysaccharide capsule, and capsules produced by this mutant were smaller compared to the WT and *vtc4*∆ strains (Fig. 5A and B). SEM analysis of the capsular ultrastructure revealed that the double mutant displayed capsular fibers that were more spread apart and scattered compared to WT and *vtc4*∆ mutant cells (Fig. 5A). The *vtc4*∆ *epp1*∆ *xpp1*∆ mutants also elaborated smaller capsules compared to the WT and the *vtc4*∆ mutant (Fig. 5B). These mutants also showed perturbed capsular ultrastructure (Fig. 5A). The absence of polyP in the triple mutant suggests that the polyphosphatases have other influences on capsule. A contribution of polyP is also possible because previous studies revealed that the molecule is required for correct capsular assembly, and cells defective in polyP synthesis show an altered capsular architecture characterized by thicker polysaccharide fibers (Fig. 5A) (41).

We also discovered that the double and triple mutants showed reduced production of melanin on a medium containing L-DOPA as a substrate, compared to WT and the *vtc4*∆ mutant (Fig. 5C). This cell surface change, along with the capsule changes, prompted us to test the ability of the mutants to respond to membrane and cell wall stress agents such as SDS, Congo Red, CFW, and caffeine. We discovered that all of the mutants were more sensitive to SDS, indicating a potential need for a balanced level of polyP to support membrane integrity (Fig. 5D). Surprisingly, all of the mutants grew normally on YPD supplemented with caffeine, CFW, and Congo Red (Fig. S7). However, staining with calcofluor white (CFW) revealed reduced chitin content for both the *xpp1*∆*epp1*∆ and *vtc4*∆*epp1*∆*xpp1*∆ mutants compared to WT and the *vtc4*∆ mutant (Fig. 5E). Overall, these results indicate that the polyphosphatases contribute to the elaboration of key virulence factors including capsule and melanin, thus providing mechanistic clues to explain the attenuated virulence.

### Xpp1 and Epp1 influence mitochondrial morphology and stress resistance

**Fig 6:**
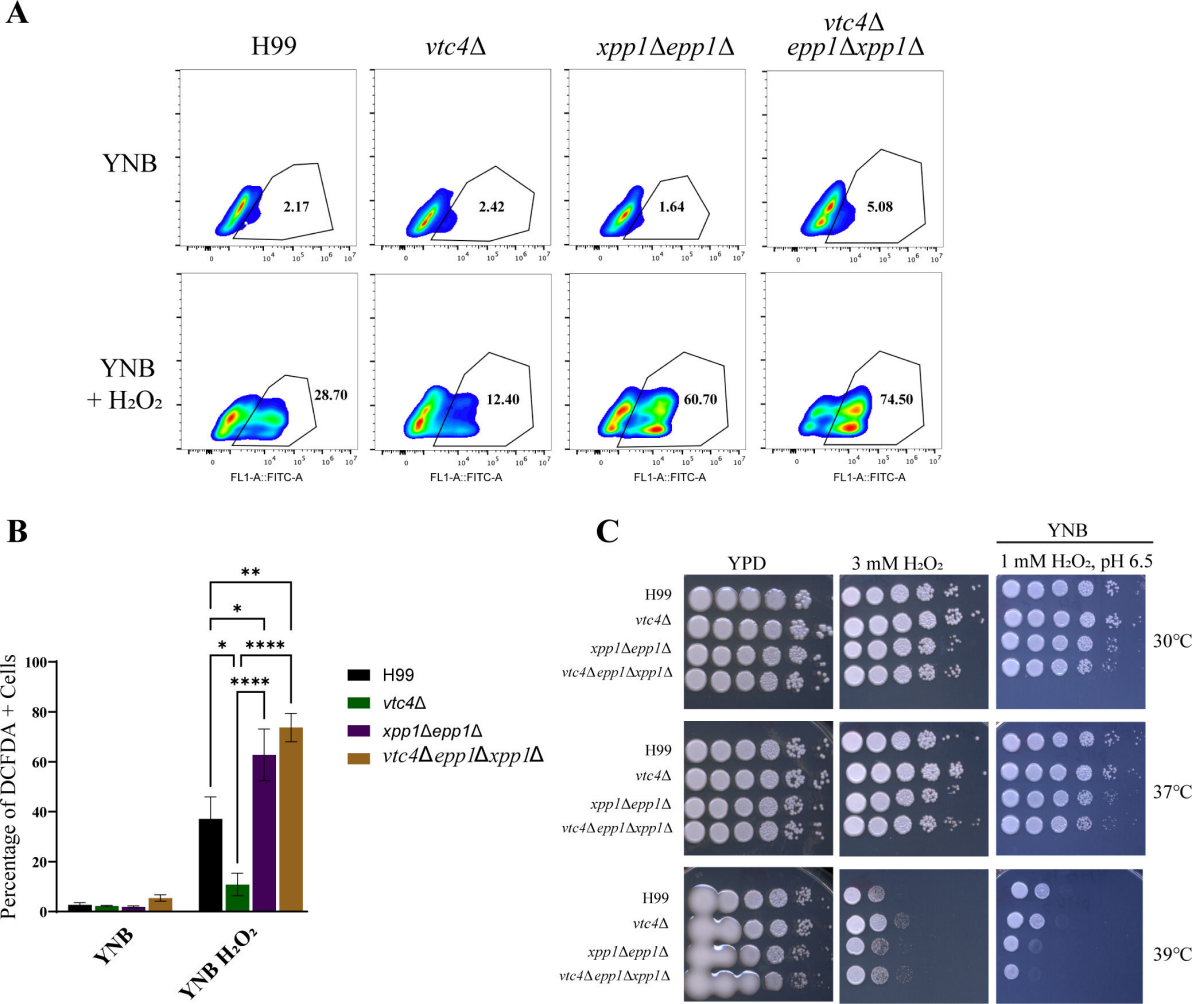
Defects in polyP mobilization confer sensitivity to reactive oxygen species stress. (**A and B**) Flow cytometry analysis of intracellular ROS levels for WT, *vtc4*∆, *xpp1*∆*epp1*∆, or *vtc4*∆*epp1*∆*xpp1*∆ (∆∆∆) strains. Indicated strains were grown in YPD overnight at 30°C, transferred to yeast nitrogen base (YNB) liquid medium for 3–4 hours, and log cells were then treated with or without H_2_O_2_ (1 mM) for 1 hour at 30°C. ROS levels were measured by staining the cells with ROS indicator dye 2′,7ʹ-Dichlorofluorescein Diacetate (DCFDA, 16 µM) for 1 hour. (**C**) Indicated strains were serially diluted and spotted onto solid YPD or YNB agar with or without H_2_O_2_. The plates were then incubated at 30°C, 37°C, or 39°C for 2–4 days before being photographed. Data are presented as mean ± SEM and representative of at least four independent experiments. Significance indicated as *P < 0.05; **P < 0.01; ***P < 0.001; ****P < 0.0001; one-way ANOVA.

A recent study linking polyP in mitochondria to the generation of reactive oxygen species (ROS) in mammalian cells prompted us to analyze ROS and mitochondria-related phenotypes for our mutants (42). We first assessed the accumulation of intracellular ROS using 2ʹ,7ʹ-dichlorofluorescein diacetate (DCFDA). Both the *xpp1*∆ *epp1*∆ and *vtc4*∆ *epp1*∆ *xpp1*∆ mutants showed elevated levels of intracellular ROS compared to WT while the *vtc4*∆ mutant showed reduced levels (Fig. 6A and B). We next assessed the ability of the mutants to respond to oxidative stress agents such as hydrogen peroxide (H_2_O_2_), paraquat, plumbagin, and *tert*-butyl hydroperoxide (tBuOOH). We discovered that the double and triple mutants were sensitive to H_2_O_2_ at elevated temperatures and that the *vtc4*∆ mutant behaved as WT (Fig. 6C). All of the mutants grew normally on media supplemented with other ROS stress agents (Fig. S8A)

**Fig 7:**
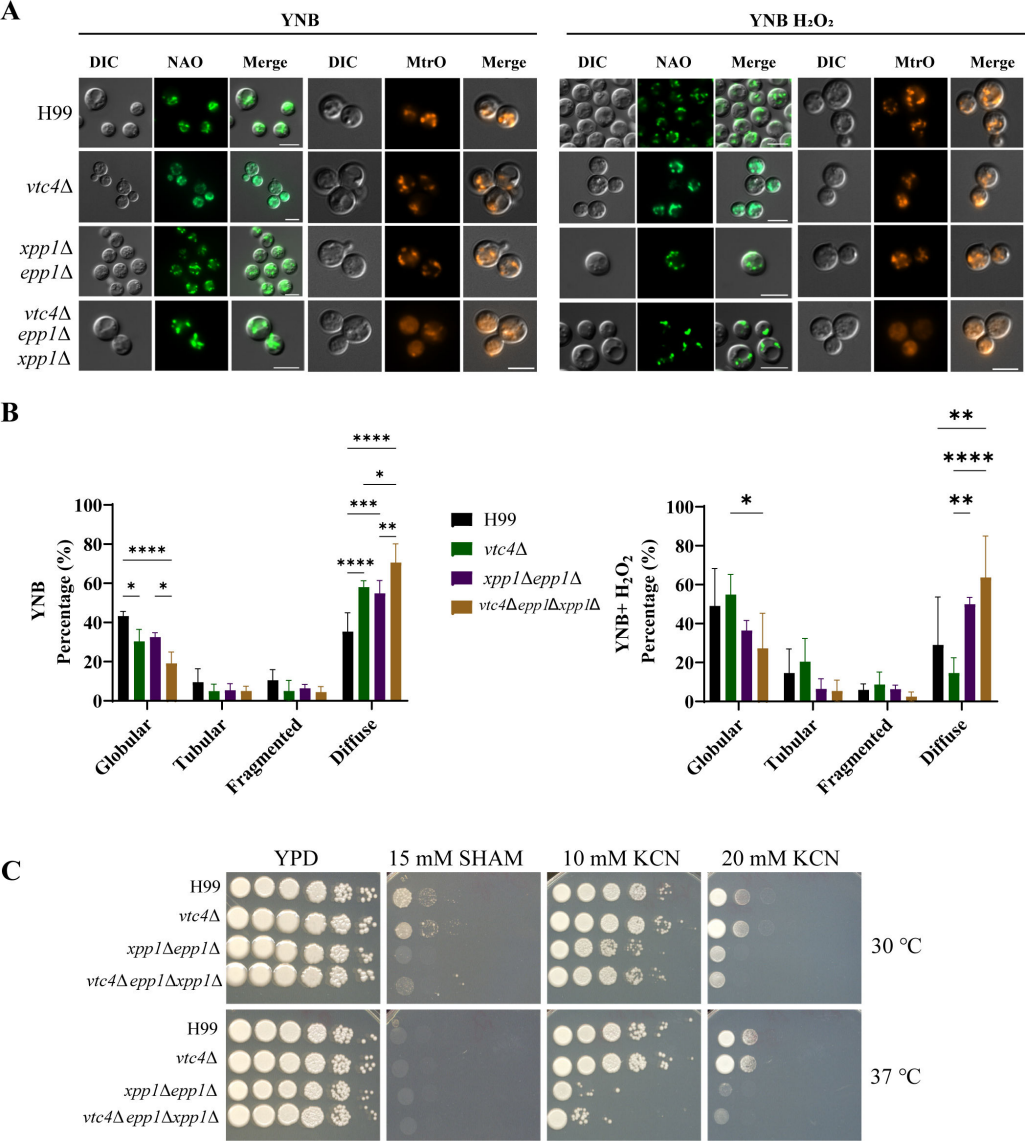
Defects in polyP mobilization impact mitochondrial morphology. (**A**) Representative widefield fluorescence micrographs showing mitochondrial morphologies in WT, *vtc4*Δ, *xpp1*∆*epp1*∆, or *vtc4*∆*epp1*∆*xpp1*∆ strains. Indicated strains were stained with the mitochondria-specific dyes, MitoTracker Orange CMTMRos (200 nM), or nonyl acridine orange (NAO, 200 ng/µL) for 30 minutes. Scale bar 5 µm. Data representative of three independent experiments representing at least 100 cells per treatment. (**B**) Analysis of the indicated strains showing the percentage of cells with different mitochondrial morphologies. Cells previously grown overnight in YNB were treated with or without H_2_O_2_ (1 mM) at 30°C for 1 hour before being stained with the mitochondria label nonyl acridine orange (NAO, 200 nM). The results represent the mean from three independent experiments ± standard deviations representing at least 100 cells for each treatment. Statistical significance was determined by two-way ANOVA followed by Tukey’s multi-comparison *post hoc* test (*P < 0.05; **P < 0.01; ***P < 0.001; ****P < 0.0001). (**C**) Indicated strains were serially diluted and spotted onto solid YPD agar with or without mitochondrial stress agents, SHAM and KCN. The plates were then incubated at 30°C for 2–4 days before being photographed.

**Fig 8:**
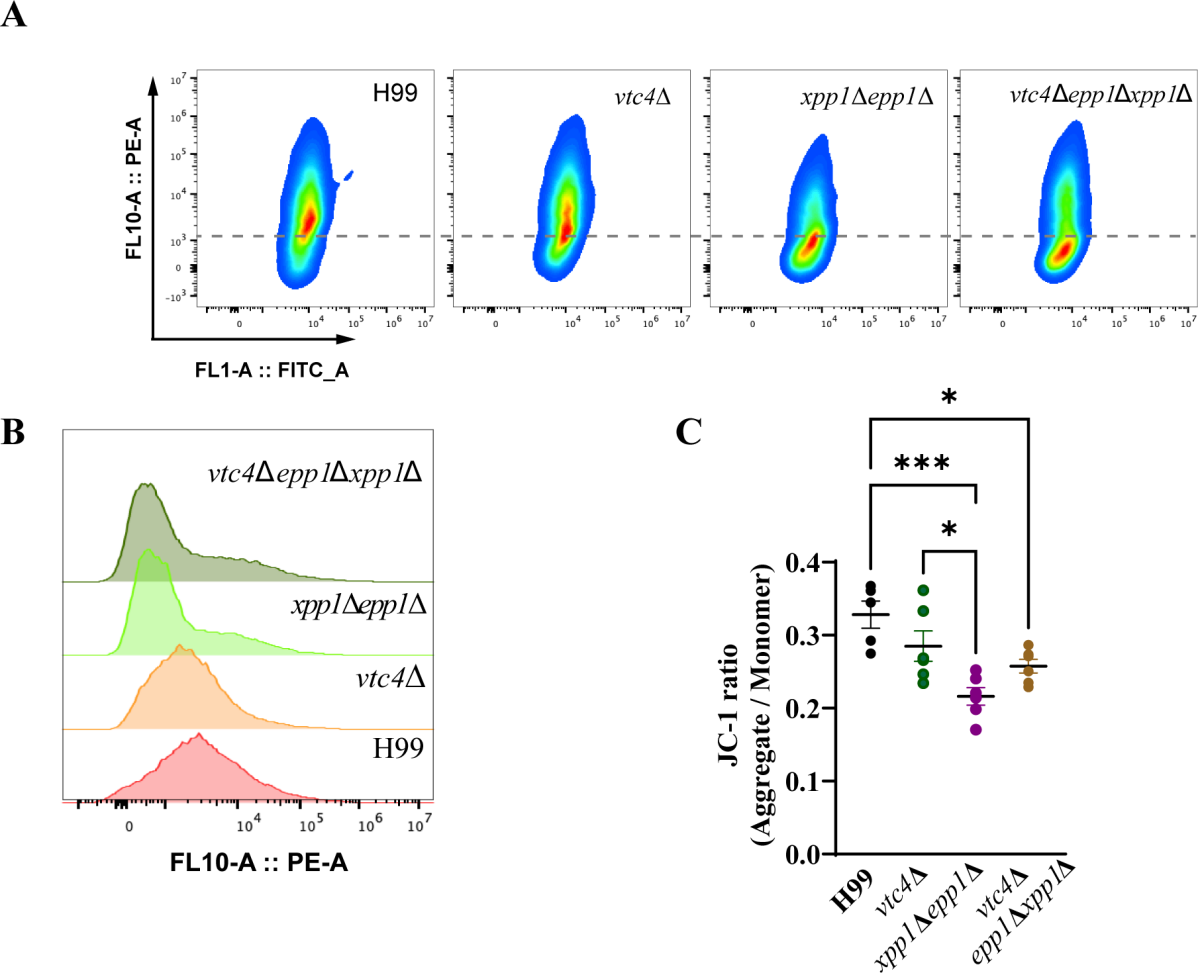
Loss of endo- and exopolyphosphatases impacts mitochondrial membrane potential. (**A**) Flow cytometry was used to analyze changes in mitochondrial membrane potential in the indicated strains. Cells were grown to log phase in YNB at 30°C and stained with the mitochondrial dye JC-1 for 30 minutes at 30°C before measurement. (**B**) Histogram of representative samples from the analysis in (**A**) showing fluorescence intensities of JC-1 aggregates. (**C**) JC-1 fluorescence ratios (aggregates/monomers) of the specified strains were analyzed by flow cytometry. Data represent the mean from three biological replicates across two independent experiments ± standard error of the mean (SEM). Statistical significance was determined by one-way ANOVA followed by Tukey’s multi-comparison *post hoc* test (*P < 0.05; ***P < 0.001).

Previous studies have highlighted the significance of mitochondria in the adaptation of fungal pathogens to diverse stress conditions (43). Mitochondrial morphology, for example, has been linked to the response to host conditions during *C. neoformans* infections (44, 45). In light of the observed sensitivities of the mutants to H_2_O_2_ (Fig. 6), we focused on examining potential changes in mitochondrial morphology in the deletion mutants with and without exposure to oxidative stress. Specifically, we employed both MitoTracker Orange and nonyl acridine orange (NAO) to investigate how oxidative stress impacts mitochondrial morphology in the mutants. These dyes allowed us to visualize both active mitochondria, based on membrane potential, and mitochondria independent of their membrane potential. In the absence of oxidative stress, all of the mutants had a reduced percentage of mitochondria with a globular morphology and a higher percentage of diffuse mitochondrial staining compared with the WT strain. Upon treatment with H_2_O_2_, the reduction in mitochondria with a globular morphology was most marked for the triple mutant, and the double and triple mutants showed higher percentages of diffuse-staining mitochondria (Fig. 7A and B). This result prompted an assessment of the ability of the mutants to respond to mitochondrial stress provoked by growth on media supplemented with the electron transport chain inhibitors rotenone (complex I), antimycin A, myxothiazol (complex III), SHAM (alternative oxidase), or KCN (complex IV). Notably, the double and triple mutants (but not the *vtc4*Δ mutant) were sensitive to SHAM and KCN but grew normally on media supplemented with antimycin A, myxothiazol, and rotenone (Fig. 7C; Fig. S8B). Finally, as an additional test of mitochondrial health, we examined membrane polarization by staining with the dye JC-1 and performing flow cytometry (Fig. 8). We found that the double and triple mutants had reduced membrane polarization compared to the WT and *vtc4*Δ strains, as demonstrated by a lower ratio of aggregated to monomeric dye. Together, these results indicate the loss of the polyphosphatases negatively impacts mitochondrial health.

## DISCUSSION

**Fig 9:**
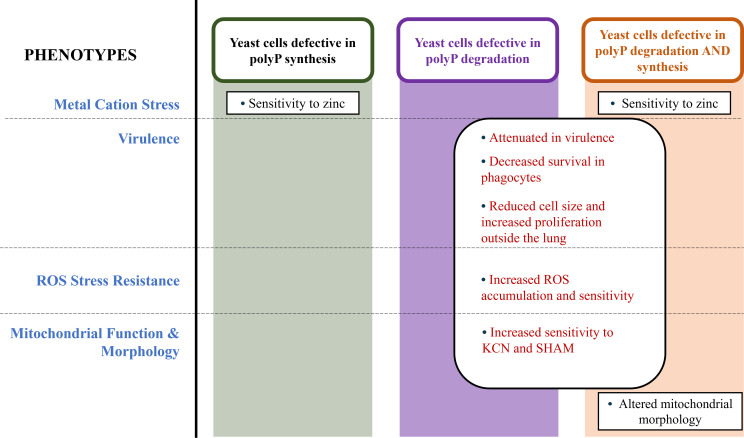
Summary diagram of the phenotypes observed for mutants deficient in polyP synthesis (*vtc4*∆), polyP degradation (*xpp1*∆*epp1*∆), and both polyP degradation and synthesis (*vtc4*∆*xpp1*∆*epp1*∆). Loss of polyP synthesis confers sensitivity to zinc metal in both *vtc4*Δ and triple mutants; both mutants are defective in polyP synthesis and accumulate no detectable polyP. Defects in virulence, ROS stress resistance, and mitochondrial function are shared by mutants defective in polyP degradation. Specifically, both the double and triple mutants display phenotypes distinct from the vtc4∆ mutant and, therefore, suggest polyP-independent functions, including attenuated virulence, decreased survival in phagocytes, reduced cell size, elevated ROS accumulation and sensitivity, and increased susceptibility to ETC inhibitors, KCN, and SHAM. Interestingly, only the triple mutant exhibits altered mitochondrial morphology.

In this study, we made use of mutants lacking the polyphosphatases Epp1 and Xpp1 to examine the influence of hyperaccumulation of polyP on cryptococcal virulence. We found that the double mutant was attenuated for virulence and provoked an altered immune response in mice. Given that our previous study revealed no virulence defect for the *vtc4∆* mutant lacking polyP (32), we specifically tested the role of polyP in the *xpp1*∆ *epp1*∆ double mutant by also deleting the *VTC4* gene. This triple mutant was also attenuated for virulence, leading to the conclusion that the polyphosphatases may contribute to disease via mechanisms independent of polyP. To examine these proposed additional contributions and other potential influences of polyP hyperaccumulation, we compared the shared and distinct virulence-related phenotypes of *vtc4∆, xpp1*∆ *epp1*∆, and *vtc4*∆ *epp1*∆ *xpp1*∆ mutants. A number of shared phenotypes for the double and triple mutant were distinct from the *vtc4∆* mutant and therefore suggest polyP-independent functions (Fig. 9). These phenotypes included reduced survival in bone marrow-derived macrophages, altered capsule size and architecture, reduced melanin and chitin accumulation, elevated ROS accumulation and sensitivity, and increased sensitivity to alternative oxidase (SHAM) and ETC (KCN) inhibitors. The double and triple mutants also shared the interesting phenotype of small cell size and greater accumulation in organs beyond the lung compared with the WT strain. Sensitivity to zinc was the notable shared phenotypes for the *vtc4∆* and *vtc4*∆ *epp1*∆ *xpp1*∆ mutants, presumably due to loss of sequestration by polyP, and our previous study documented the association of polyP accumulation with zinc resistance (32). Interestingly, the triple mutant also displayed the distinct phenotype of diffuse mitochondrial staining, which may indicate a synthetic interaction resulting from the loss of Vtc4 and the polyphosphatases. It is possible, for example, that the phenotype results from the combined loss of polyP and the loss of the polyP-independent activities of Xpp1 and Epp1. We also noted that all three mutants shared sensitivity to SDS, perhaps indicating the need for a balanced level of polyP to support membrane integrity.

Our observation of a distinct phenotype for the triple mutant with regard to mitochondrial morphology suggests the potential for combined roles for polyP and the polyphosphatases in mitochondrial function. There is considerable evidence that polyP impacts mitochondrial functions in other organisms. In mammalian cells, polyP co-localizes to mitochondria and plays a crucial role in maintaining cellular bioenergetics as well as regulating oxidative phosphorylation and glycolysis (42, 46, 47). PolyP is also known to regulate the calcium concentration in mitochondria which, in turn, is needed for ATP production (47). Interestingly, our polyphosphatase mutants were sensitive to KCN and SHAM, inhibitors of cytochrome oxidase and alternative oxidase, respectively. This finding suggests that enzymes involved in polyP breakdown have alternative functions in the later part of the electron transport chain and may be more directly involved in ATP synthesis. A recent study investigating mitochondrial bioenergetics revealed that depletion of mitochondrial polyP results in increased oxidative stress conditions due to a dysregulation of oxidative phosphorylation, and these conditions are characterized by increased intracellular H_2_O_2_ levels (42). We suspect that the loss of Xpp1 and Epp1 in *C. neoformans* leads to a similar outcome as mitochondrial polyP may not be mobilized for regulation of redox homeostasis. This difference, coupled with perturbed mitochondrial function and morphology, might explain elevated ROS levels in *C. neoformans* mutants deficient in polyphosphatases. An additional potential connection between the polyphosphatases and mitochondrial function is suggested by a recent analysis of interacting partners for Vps39, a component of the HOPS complex for endomembrane trafficking and the vCLAMP membrane contact site (MCS) between mitochondria and the vacuole (48). The analysis revealed a key interaction between Vps39 and the Tom40 mitochondrial protein as part of the vCLAMP MCS. Intriguingly, the polyphosphatase Ppn1 was also identified as an additional interactor for Vps39, thus hinting at potential connections with endomembrane trafficking and mitochondrial function that require further investigation.

Our understanding of how polyP synthesis and degradation intersect with virulence in fungal pathogens is limited. To our knowledge, immune responses to fungal polyP have not been studied in detail, and the influence of polyP on the immune system is quite complex and not fully understood. It is believed that polyP polymers of different chain lengths activate distinct signaling pathways and have diverse mechanisms of action (49). A recent study investigating the role of the Ppn1 and Ppx1 polyphosphatases in *C. albicans* revealed that polyP mobilization is required for DNA replication, and blocking polyP mobilization results in impaired cell division and formation of large pseudohypha-like cells (38). In addition, polyP mobilization is required for the virulence of *C. albicans* in both the wax moth *Galleria mellonella* and murine models (38). Loss of the polyphosphatases also blocks the activation of Pho4, the key regulator of genes for phosphate acquisition. Interestingly, a *vtc4*Δ mutant in *C. albicans* also had impaired virulence in the wax moth model in support of a contribution of polyP. A similar situation exists for the fungal phytopathogen *Ustilago maydis* where Vtc4 is required for full virulence on maize seedlings but not for sporulation (34). Thus, the influence of polyP on virulence appears to vary depending on the fungal pathogen under investigation.

In this regard, the polysaccharide capsule is a unique virulence attribute of *C. neoformans* (11). Our previous studies indicated a role for polyP at the cell surface in that Vtc4 was required for proper capsular assembly (41). Expanding upon this finding, we found that the mutants lacking Vtc4, Xpp1, and Epp1, or all three proteins, exhibited abnormal polysaccharide capsular fibers. In particular, the double and triple mutants had fibers that were characterized by greater dispersion and scattered distribution compared to the WT strain or the *vtc4*Δ mutant. This discovery suggests a novel function for polyphosphatases in the elaboration of the cryptococcal polysaccharide capsule that, along with a melanin defect, could potentially contribute to attenuated virulence. We also demonstrated that cells of the double and triple mutants were smaller in cell size *in vivo* and showed enhanced extrapulmonary dissemination over time. These observations are consistent with the findings of Denham et al. (40). The presence of small or “micro” cells is also associated with decreased CD4 T-cell counts (50), which is consistent with our results. Large cells are known to be resistant to oxidative and nitrosative stress and show reduced extrapulmonary dissemination (51).

Studies with bacterial pathogens are shedding light on how polyP not only influences cellular functions but also modulates the host immune response. In *Mycobacterium tuberculosis,* a mutant lacking exopolyphosphatases is significantly reduced in the expression of virulence genes, and accumulation of polyP prevents the establishment of infection in guinea pigs (52). *M. tuberculosis* mutants defective in polyP mobilization also show significantly reduced growth and survival in macrophages (52, 53). High levels of bacterial polyP also serve to deter phagocytosis by macrophages and neutrophils and contribute to the inhibition of phagosome acidification in phagocytes (54). PolyP can also inhibit the recruitment of macrophages into tissue (55). In mammalian cells, it is hypothesized that polyP acts as a signal that impacts leukocyte recruitment, function, and proliferation (53, 56). PolyP is also known to contribute to the proinflammatory effect of activated platelets and has roles in blood coagulation (57). A recent study reported that short-chain polyP potentiates the activity of neutrophils and stimulates the release of neutrophil extracellular traps (NETs) (58). By contrast, our analysis indicates that mice infected with the *xpp1*∆*epp1*∆ double mutant had significantly decreased numbers of CD45+ cells, neutrophils, and eosinophils in lung tissue at 7 dpi. The diminished presence of CD45+ cells in the lung may stem from an initial lower fungal burden within lung tissue, as fewer yeast cells might elicit weaker immune responses. In addition, mice infected with the *xpp1*∆*epp1*∆ mutant exhibit reduced IL-4 levels compared to those infected with the WT strain. IL-4 plays detrimental roles in fungal immunity by polarizing macrophages to an alternatively activated phenotype, promoting the proliferation of intracellular pathogens (59). This suggests that infection with the double mutant promotes a protective Th1-type response, potentially facilitating more effective pathogen clearance, especially at earlier time points. Overall, the *xpp1*Δ*epp1*Δ mutant stimulated a weaker immune response compared to the wild type.

It is currently unclear why the mice infected with *xpp1*Δ*epp1*Δ and *vtc4*∆*epp1*∆*xpp1*∆ mutants ultimately succumbed to the infection. It is possible that mutants struggled to establish a robust infection during earlier timepoints and needed time to adapt to the host environment. Evidence for this idea comes from the finding that mice infected with the *xpp1*∆*epp1*∆ mutant showed reduced fungal burdens at day 7. This could also account for the gradual decline in murine weight and the slow but steady progression of the disease. We speculate that the *xpp1*∆*epp1*∆ mutant stimulates a weaker immune response characterized by reduced numbers of CD45+ cells, neutrophils, eosinophils, CD4+ T cells, and CD8+ T cells in the BALB/c mice at earlier infection timepoints which allows these mutants to survive in the lung environment and cause enhanced disease later in the infection period. It is also likely that a defect in uptake by macrophages combined with a diminished presence of eosinophils permits the *xpp1*Δ*epp1*Δ to survive in the extracellular space and proliferate in the lung tissue at later infection timepoints. It is interesting that despite no differences in lung monocyte numbers between WT and double mutant strains, mice infected with the *xpp1*Δ*epp1*Δ mutant have significantly reduced monocyte-derived macrophages. This observation hints at a possible block in the differentiation of monocytes into monocyte-derived macrophages, warranting further investigation.

In summary, we find that the polyphosphatases Epp1 and Xpp1 influence virulence in the murine model of cryptococcosis by mechanisms that are at least partially independent of polyP accumulation. We propose that attenuated virulence results from reduced survival in macrophages, susceptibility to reactive oxygen species perhaps resulting from impaired mitochondrial function, and reduced formation of the critical virulence factors capsule and melanin. Additional work is needed to understand the polyP-dependent and -independent functions, including potential regulatory roles in the expression of genes in the Pho regulon and connections with inositol polyphosphates (IPs) and inositol pyrophosphates (PP-IPs) (25, 35, 37). The evaluation of interactions of the polyphosphatases with other proteins should also shed light on additional functions to explain the roles of the enzymes beyond polyP mobilization.

## MATERIALS AND METHODS

### Strains, plasmids, and media

The *C. neoformans* var. *grubii* strain H99 (serotype A) and derived mutants were used in the experiments. *C. neoformans* deletion mutants lacking Xpp1, Epp1, or both proteins (Table S1) were from our previous study (32). Strains were maintained on YPD-rich medium (1% yeast extract, 2% peptone, 2% dextrose, and 2% agar). The hygromycin resistance cassette originated from pJAF15. YPD plates containing hygromycin (200 µg/mL) were used to select transformants. Unless specified otherwise, all chemicals were obtained from Sigma-Aldrich. Iron-chelated dH_2_O was prepared by passage through a column containing Chelex-100 resin (BIORAD Chelex-100) and used in the preparation of low-iron media (LIM) (0.5% glucose, 38 mM L-asparagine, 2.3 mM K_2_HPO_4_, 1.7 mM CaCl_2_•H_2_O, 0.3 mM MgSO_4_•7H_2_O, 20 mM HEPES, 22 mM NaHCO_3_, 1 mL of 1,000× salt solution (0.005 g/L CuSO_4_•5H_2_O, 2 g/L ZnSO_4_•7H_2_O, 0.01 g/L MnCl_3_•4H_2_O, 0.46 g/L sodium molybdate, 0.057 g/L boric acid in iron-chelated dH_2_O adjusted to pH 7.4 with 0.4 mg/L sterile thiamine added post-filtering) as described previously (60).

### Construction of deletion mutants

The *vtc4*Δ*epp1*Δ*xpp1*Δ (ΔΔΔ) triple deletion mutant was constructed by homologous recombination using gene-specific cassettes amplified via a three-step overlapping PCR method utilizing the primers detailed in S2 Table (61). The resistance marker for hygromycin (HYG) was amplified by PCR using primers ∆∆∆−2 and ∆∆∆−5 and the plasmid pJAF15, respectively, as the template. Primers ∆∆∆−1 and ∆∆∆−3, as well as ∆∆∆−4 and ∆∆∆−6, were employed to amplify the surrounding sequences of the *vtc4* gene, while primers ∆∆∆−1 and ∆∆∆−6 were utilized to amplify the deletion construct of the gene, incorporating the resistance marker. The construct was introduced into the *epp1*Δ*xpp1*Δ−4 strain by biolistic transformation, as described previously (62). Colony PCR was performed to identify positive transformants using primers ∆∆∆−7N, ∆∆∆−8N, HYGL, and HYGR. Primers used in mutant screening and verification are summarized in S2 Table. Two independent mutants were used for all experiments unless stated otherwise.

### Mice

Female BALB/c or C57BL/6 mice, 4- to 6-week old, were obtained from Charles Rivers Laboratories (Pointe-Claire, Quebec, Canada) and housed in the Modified Barrier Facility at the University of British Columbia (UBC). Mice were housed in sterilized cages with microisolator cage tops, maintaining specific pathogen-free conditions. Clean food and water were provided *ad libitum*. Experiments were conducted with the protocols approved by the University Animal Care Committee in accordance with the Canadian Council of Animal Care guidelines for ethical animal research.

### Murine infection and assessment of virulence

Fungal cells were cultured in 5 mL YPD at 30°C overnight, washed three times with physiological saline, and resuspended in physiological saline (pH 7.4). A cell suspension of 2 × 10^5^ cells in 50 µL was intranasally instilled. The status of the mice was monitored once per day post-inoculation. Mice reaching the experimental endpoint of 15% wt loss were euthanized by an overdose of isoflurane followed by carbon dioxide asphyxiation. Differences in virulence were statistically assessed using log-rank tests. For determination of fungal burden, lungs, livers, spleens, kidneys, and livers were excised, weighed, and homogenized in 2 volumes of 1× phosphate-buffered saline using a MixerMill (Retsch, Cole-Parmer, Montreal, Canada). Serial dilutions of the homogenates were plated on YPD plates containing 50 µg/mL chloramphenicol, and colony-forming units were counted after 48 hours at 30°C.

### Cytometric Bead Array (CBA) Assay

Lungs were excised and collected in PBS containing a 2× cOmplete, EDTA-free protease inhibitor cocktail (Roche). Lungs were homogenized using a mixer mill. Supernatants of homogenates were collected, and cytokines in the samples were analyzed using the BD cytometric Th1/Th2/Th17 kit (BD Biosciences). The kit was used for the simultaneous detection of mouse interleukin-2 (IL-2), interleukin-4 (IL-4), interleukin-6 (IL-6), interferon-γ (IFN-γ), tumor necrosis factor (TNF), interleukin-17A (IL-17A), and interleukin-10 (IL-10) in a single sample. The operations were formed according to the manufacturer’s instructions. Beads coated with seven specific capture antibodies were fixed. Subsequently, 50 µL of the mixed captured beads, 50 µL of the unknown lung supernatant or standard dilution, and 50 µL of phycoerythrin (PE) detection reagent were added to each assay tube and incubated for 2 hours at room temperature in the dark. The samples were washed once with 5 mL of wash buffer and centrifuged. The bead pellet was resuspended in 300 µL buffer. Samples were measured on the CytoFLEX S (Beckman Coulter) Flow Cytometer equipped with four laser lines (405 nm, 488 nm, 561 nm, and 633 nm) fitted with filters FITC (525/40), PE (585/42), ECD (600/20). Individual cytokine concentrations were indicated by their fluorescent intensities. Cytokine standards were serially diluted to facilitate the construction of calibration curves, necessary for determining the protein concentrations of test samples.

### Histology

Lungs were harvested and fixed overnight in 10% buffered formalin. Samples were dehydrated, paraffin-embedded, sectioned, and stained with either hematoxylin and eosin (H&E) or Movat’s Pentachrome. Images were acquired using a Nikon ECLIPSE Ti2 inverted microscope equipped with a Nikon DS-Ri2 high-resolution full-frame sensor microscope camera (Nikon Instruments Inc.). For visualization and image processing, NIS-Elements Viewer software was used.

### Bronchoalveolar lavage (BAL)

Mice were euthanized through an isoflurane overdose, followed by carbon dioxide asphyxiation. Alveolar cells were collected via BAL using catheterization of the trachea and washed three times, with each wash consisting of 1 mL 1× PBS and 2 mM EDTA. The cells were then pelleted, treated with RBC lysis buffer (10 mM Tris, 0.84% NH_4_Cl), and prepared for flow cytometry.

### Tissue processing and analysis of immune cells by flow cytometry

Lungs were perfused with 15–20 mL of ice-cold 1× PBS (pH 7.4) until the tissue turned white. Perfused lungs were excised, minced, and enzymatically digested in 5 mL of 0.7 mg/mL collagenase IV (Worthington), 50 µg/mL DNAse I (Worthington) in RPMI 1640 for 1 hour at 37°C. Dissociated cells and tissue were passed through a 70 µm strainer, red blood cells were lysed with RBC lysis buffer (10 mM Tris, 0.84% NH_4_Cl) for 5 min at room temperature, and the remaining homogenate was passed through a 35 µm strainer to generate a single-cell suspension. Isolated single cells were then incubated with 2.4G2 tissue culture supernatant to block Fc receptors and immunolabeled for cell surface antigens for 20 minutes at 4°C in flow cytometry buffer (PBS, 2% bovine serum albumin, 2 mmol/L EDTA). Multiparameter assessment was performed using the Attune NxT Flow cytometer (Thermo Fisher Scientific) and data were analyzed with FlowJo software (TreeStar). The following antibodies against mouse antigens were used for flow cytometry: CD45 (30-F11), CD24 (M1/69), CD11c (N418), CD11b (M1/70), CD64 (X54-5/7.1), Ly6C (HK1.4), Ly6G (1A8), CD4 (RM4-5) from BioLegend; NK1.1 (PK136), SiglecF (1RNM44N), MHCII (M5/114.15.2) from eBioscience; CD3 (145–2C11), CD8 (53–6.7), CD19 (1D3) from AbLab (UBC). The Zombie Aqua fixable viability kit was used to identify live cells (BioLegend). Cell numbers were calculated using counting beads (123 count eBeadsTM, eBioscience/Thermo Fisher Scientific). Data were analyzed in GraphPad Prism 10.0, and a two-tailed unpaired Student *t* test was used when comparing different biological samples.

### Stress and drug response assays

Exponentially growing cultures of WT or mutant strains were washed, diluted to an initial concentration of 1 × 10^8^ cells/mL in H_2_O, diluted 10-fold serially, and 5 µL of each dilution was spotted onto YPD or YNB plates supplemented with the following compounds: 2.5 mM ZnCl_2_, 3.0 mM NiSO_4_, 1.875 mM MnSO_4_, 0.5 M CaCl_2_, 1 mg/mL Caffeine, 1 mg/mL CFW, 1% Congo Red, 0.5% SDS, 1 mM or 3 mM H_2_O_2_, 15 mM Salicylhydroxamic acid (SHAM), 10 mM or 20 mM KCN, 5 µg/mL Antimycin A, 5 µM Myxothiazol, 50 µg/mL Rotenone, 0.25 mM Paraquat dichloride, 25 µM Plumbagin (Cedarlane Labs), or 1 mM tert-Butyl hydroperoxide (tBuOOH). Plates were incubated for 2–5 days at 30°C, 37°C, or 39°C and photographed.

### Macrophage uptake and survival assays

Bone-marrow-derived macrophages (BMDMs) were generated by culturing bone marrow cells with L-929 cell conditioned medium (LCCM) for 6 days. The cultivation of macrophages was performed in DMEM supplemented with LCCM, 20% bovine serum albumin, 1 mM Na-pyruvate, and 2 mM L-glutamine at 37°C, 5% CO_2_. Phagocytosis and intracellular replication of WT and mutant strains in the BMDMs were determined as previously described (63). Briefly, macrophage cells in 24-well plates were stimulated with 150 ng/mL phorbol myristate acetate (PMA) for 1 hour prior to infection. Cells of the wild-type or mutant strains were opsonized for 1 hour with the monoclonal antibody 18B7. Stimulated macrophages were infected with 2 × 10^6^ opsonized yeast cells (10:1 multiplicity of infection; yeast: macrophage) for 2 and 24 hours at 37°C, 5% CO_2_. Non-internalized yeast cells were washed off three times with 1× PBS. To determine the number of internalized yeast cells, macrophages were lysed with sterile distilled H_2_O and lysates were plated on YPD agar for CFU counts at both 2 and 24 hours.

BMDMs were seeded in eight-well chamber slides in DMEM medium and allowed to adhere firmly overnight. The next day, macrophage cells were washed and maintained in serum-free DMEM for infection with opsonized yeast cells. For the analysis of macrophage uptake, live differential interference contrast (DIC) images were taken 2 hours post-infection, and at least 300 macrophages were counted per strain for each experiment. For the analysis of intracellular survival, non-internalized yeast cells were washed off three times with 1× PBS, and live DIC images were taken 24 hours post-infection.

### Fluorescence microscopy

For chitin detection, cells were grown overnight in YPD, washed twice in 1× PBS, diluted to an OD_600_ nm of 1, and stained with calcofluor white (CFW) in PBS for 15 minutes at room temperature in the dark.

For the analysis of mitochondria morphology, cells were grown in YNB overnight at 30°C. Precultures were washed in minimum media (YNB) twice and resuspended (4 OD) in YNB with or without H_2_O_2_ (1 mM). Cells were incubated at 30°C for 1 hour at 200 rpm. Subsequently, cells were collected and washed twice with PBS before staining with the mitochondria dyes Mitotracker Orange CMTMRos (Invitrogen, 200 nM) or Acridine Orange 10-nonyl bromide (Invitrogen, 200 nM) for 30 min at 30°C in the dark. After staining, the cells were collected and washed with PBS and kept on ice before microscopy observations. Differential interference contrast (DIC) and fluorescence imaging were performed with a wide-field fluorescence microscope (Zeiss Axiovert 200) coupled with a CMOS camera (ORCA-Flash4.0 LT; Hamamatsu Photonics) along with a 100× oil immersion lens objective (numerical aperture [NA], 1.40). The signals from Mitotracker Orange and Acridine Orange 10-nonyl bromide dyes were captured using filter sets (Ex, 572/25 nm; Em, 629/62 nm) and (Ex, 470/20 nm; Em, 500/25 nm), respectively, with exposure times ranging from 100 to 400 ms. For cell visualization and quantification, ZEN Lite 2.3 (version 2.3.69.1000; Carl Zeiss) software was used.

### Flow cytometry measurements for intracellular ROS accumulation

Flow cytometric measurements were performed using CytoFLEX S (Beckman Coulter) Flow Cytometer equipped with four laser lines (405 nm, 488 nm, 561 nm, and 633 nm) fitted with filter FITC (525/40). The number of cells measured per experiment was set to 30,000–40,000 unless otherwise stated. For the ROS sensitivity analysis, cells were grown overnight at ~200 rpm and 30°C in YPD media and washed twice with 1× PBS. Subsequently, 0.4 OD cells were grown in 25 mL of YNB media for 3–4 hours at 200 rpm and 30°C. Cells were collected and then treated in YNB with or without hydrogen peroxide (H_2_O_2_, 5 mM) for 1 hour. After ROS treatment, cells were collected and stained with 2ʹ,7ʹ-Dichlorofluorescein Diacetate (DCFDA, 16 µM) for 1 hour at 30°C before cytometric analysis.

### Assessment of polyP accumulation

The amount of intracellular polyP was determined as previously described (5, 34). Briefly, cells were grown in YPD overnight at 30℃. RNA was extracted using a citrate buffer and bead beating in a bead mill to rupture the cells and release RNA and polyphosphate. Subsequently, 10 µg of total RNA was loaded onto a native DNA polyacrylamide gel and subjected to electrophoresis in 1 × Tris-borate-EDTA (TBE) buffer. The RNA and polyphosphate were then fixed with acetate, stained with toluidine blue O, and destained in acetate, following previously established methods. 10 µg of polyphosphate type 700 (P700; Kerafast) was used as molecular marker control.

### Assessment of virulence factor production

Elaboration of polysaccharide capsule was examined by differential interference microscopy (DIC) using a Zeiss Plan-Apochromat 100×/1.46 oil lens on a Zeiss Axioplan 2 microscope coupled with a CMOS camera (ORCA-Flash4.0 LT; Hamamatsu Photonics) after incubation for 48 hours at 30°C in defined low-iron medium (LIM). Cells were stained with India ink to visualize polysaccharide capsules. Melanin production was assessed through growth on L-3,4- dihydroxyphenylalanine (L-DOPA) plates or liquid medium containing 0.1% glucose, as previously described (64).

### Sample preparation and scanning electron microscopy

*C. neoformans* cells grown in low iron, capsule-inducing media (CIM) for 48 hours at 30°C were washed twice in 1× PBS (pH 7.4) and fixed for 1 hour in 2.5% glutaraldehyde in 0.1 M sodium cacodylate buffer (pH 7.4) at room temperature. Subsequently, the fixed cells were washed twice in 1× PBS. 20 µL of fixed cell solution was deposited on poly-L-lysine-coated coverslips (BioCoat Ref#354085). Samples were gradually dehydrated in ethanol series: 50, 60, 70, 80, 90, and three subsequent 100%. Dehydrated samples were dried using a Tousimis Samdri-795 critical point dryer before coating with 10 nm iridium using a Leica EM MED020 sputter coater. Images were acquired using a Helios NanoLab 650 dual beam SEM (Thermofisher, MA) with an Everhart-Thornley detector at a voltage between 10 and 15 kV and the working distance of 4 mm.

### Analysis of mitochondrial membrane potential by flow cytometry

Flow cytometric measurements were performed using CytoFLEX S (Beckman Coulter) Flow Cytometer equipped with four laser lines (488 nm and 561 nm) fitted with filters FITC (525/40) and PE (585/42). The number of cells measured per experiment was set to 40,000. For mitochondrial membrane potential measurements, cells were stained with the JC-1 dye (5,5′,6,6′-tetrachloro-1,1′,3,3′-tetraethylbenzimi-dazolylcarbocyanine iodide, Thermo Fisher Scientific, Waltham, MA, USA) (2.5 μM) for 30 min and 150–200 rpm. Briefly, wild-type and deletion mutant strains were grown at 30°C and ~200 rpm in YPD media for ~16 h. Subsequently, 0.3 OD_600_ cells were grown on rich media for 3 h and then collected for JC-1 staining. Data analysis and evaluation were conducted using FlowJo v10.8 Software (BD Life Sciences). Statistical analysis was performed using GraphPad Prism software.

### Statistical analysis

Statistical analysis was performed using unpaired student’s *t* tests, Kruskal-Wallis, Mann-Whitney, log-rank tests, or one-way ANOVA. All statistical tests were conducted using GraphPad Prism software.

## SUPPLEMENTAL MATERIAL

Fig. S1 iai.00072-25-s0001.pdf Histopathology of double and triple mutants.

Fig. S2 iai.00072-25-s0002.pdf Immune cell flow cytometry gating strategy.

Fig. S3 iai.00072-25-s0003.pdf Infiltration of immune cells in the lung and BAL of mice infected with *C. neoformans*.

Fig. S4 iai.00072-25-s0004.pdf The xpp1Δepp1Δ mutant provokes an altered immune response characterized by significantly reduced immune cell populations in lung tissue.

Fig. S5 iai.00072-25-s0005.pdf The vtc4Δepp1Δxpp1Δ triple deletion mutant does not have detectable polyP and exhibits sensitivity to zinc.

Fig. S6 iai.00072-25-s0006.pdf Loss of polyP synthesis and mobilization influences virulence in C57BL/6 mice.

Fig. S7 iai.00072-25-s0007.pdf Mutants deficient in polyP synthesis and mobilization are not sensitive to cell wall stress.

Fig. S8 iai.00072-25-s0008.pdf Loss of polyP synthesis and mobilization does not confer sensitivity to all ROS or ETC stress agents.

Supplemental tables iai.00072-25-s0009.pdf Tables S1 and S2.

ASM does not own the copyrights to Supplemental Material that may be linked to, or accessed through, an article. The authors have granted ASM a non-exclusive, world-wide license to publish the Supplemental Material files. Please contact the corresponding author directly for reuse.
