# Supplementary material for: Polyphosphatases have a polyphosphate-independent influence on the virulence of Cryptococcus neoformans
Source: Infect Immun. 2025 Mar 12;93(4):e00072-25. doi: 10.1128/iai.00072-25 (PMC11977306; doi:10.1128/iai.00072-25)
Supplement: Fig. S1 — Histopathology of double and triple mutants. [file iai.00072-25-s0001.pdf]

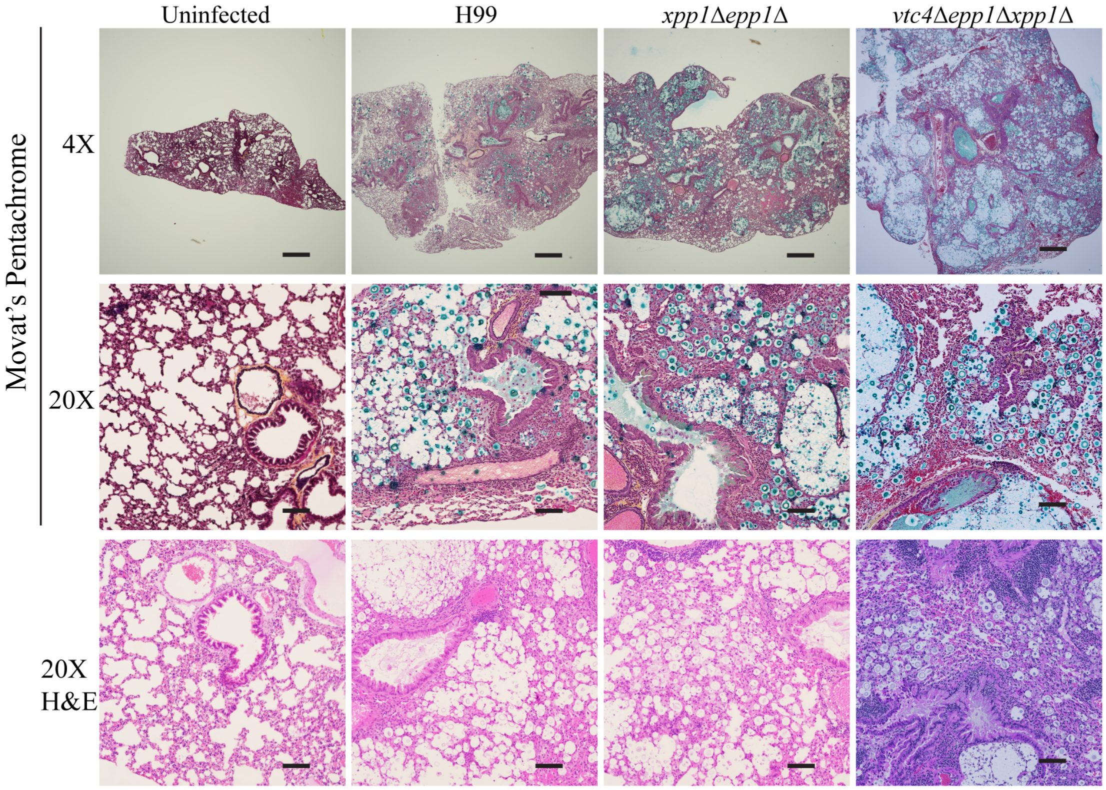

Figure S1. Histopathology of double and triple mutants. Representative histopathological micrographs of lung tissue from mice infected with WT, *xpp1Δepp1Δ* or ΔΔΔ mutants, collected at experimental endpoint and stained with H&E or Movat's Pentachrome. Scale bar: 4X = 500 µm; 20X = 100 µm.
