# Supplementary material for: Polyphosphatases have a polyphosphate-independent influence on the virulence of Cryptococcus neoformans
Source: Infect Immun. 2025 Mar 12;93(4):e00072-25. doi: 10.1128/iai.00072-25 (PMC11977306; doi:10.1128/iai.00072-25)
Supplement: Fig. S2 — Immune cell flow cytometry gating strategy. [file iai.00072-25-s0002.pdf]

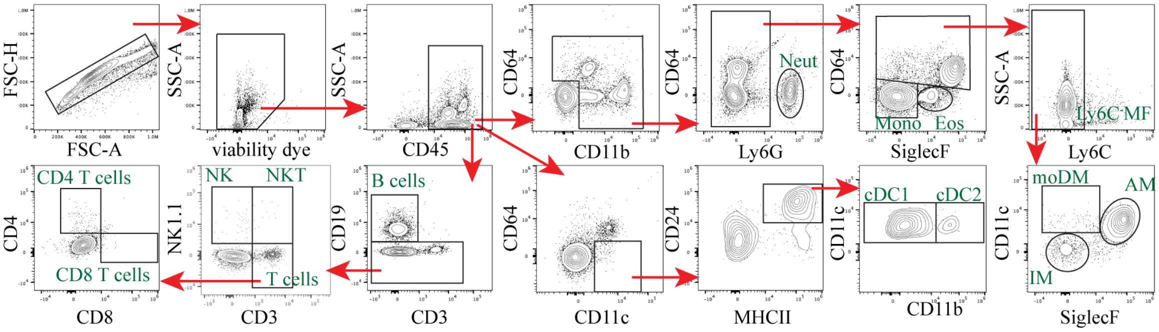

Figure S2. Immune cell flow cytometry gating strategy. Representative plots from a saline-treated lung. Doublets and debris were excluded using FSC and SSC. B cells were identified as viable CD45$^+$CD19$^+$CD3$^-$ cells. T cells were classified as viable CD45$^+$CD19$^-$CD3$^+$ cells and further separated as CD4+ and CD8+ T cells based on CD4$^+$CD8$^-$ and CD8$^+$CD4$^-$ respectively. Natural killer (NK) cells were gated as viable CD45$^+$CD19$^-$CD3$^-$NK1.1$^+$ cells. Natural killer T (NKT) cells were defined as viable CD45$^+$CD19$^-$CD3$^+$NK1.1$^+$ cells. Conventional dendritic cells type 1 (cDC1) and type 2 (cDC2) were identified as viable CD45$^+$CD24$^+$MHCII^{hi}CD11c^{hi}CD11b$^-$ and CD11b$^+$ respectively. Neutrophils (Neut) were classified as viable CD45$^+$CD11b$^+$CD64$^-$Ly6G$^+$ cells. Eosinophils (Eos) were defined as viable CD45$^+$CD11b$^+$CD64$^-$Ly6G$^-$SiglecF$^+$ cells. Monocytes (Mono) were gated as viable CD45$^+$Ly6G$^-$CD11b$^+$SiglecF$^-$CD64$^-$ cells. Ly6C$^-$CD64$^+$ macrophages (Ly6C$^-$MF) were identified as viable CD45$^+$Ly6G$^-$CD11b$^{lo/+}$CD64$^+$Ly6C$^-$ cells. Subsequently, interstitial macrophages (IM) were identified as viable CD45$^+$Ly6G$^-$CD11b$^{lo/+}$CD64$^+$Ly6C$^-$SiglecF$^-$CD11c$^-$ cells. Monocyte-derived macrophages (moDM) were classified as viable CD45$^+$Ly6G$^-$CD11b$^{lo/+}$CD64$^+$Ly6C$^-$SiglecF$^{-/lo}$CD11c$^+$ cells. Alveolar macrophages (AM) were defined as viable CD45$^+$Ly6G$^-$CD11b$^{lo/+}$CD64$^+$Ly6C$^-$SiglecF$^+$CD11c$^+$ cells.
