# Supplementary material for: Polyphosphatases have a polyphosphate-independent influence on the virulence of Cryptococcus neoformans
Source: Infect Immun. 2025 Mar 12;93(4):e00072-25. doi: 10.1128/iai.00072-25 (PMC11977306; doi:10.1128/iai.00072-25)
Supplement: Fig. S3 — Infiltration of immune cells in the lung and BAL of mice infected with C. neoformans. [file iai.00072-25-s0003.pdf]

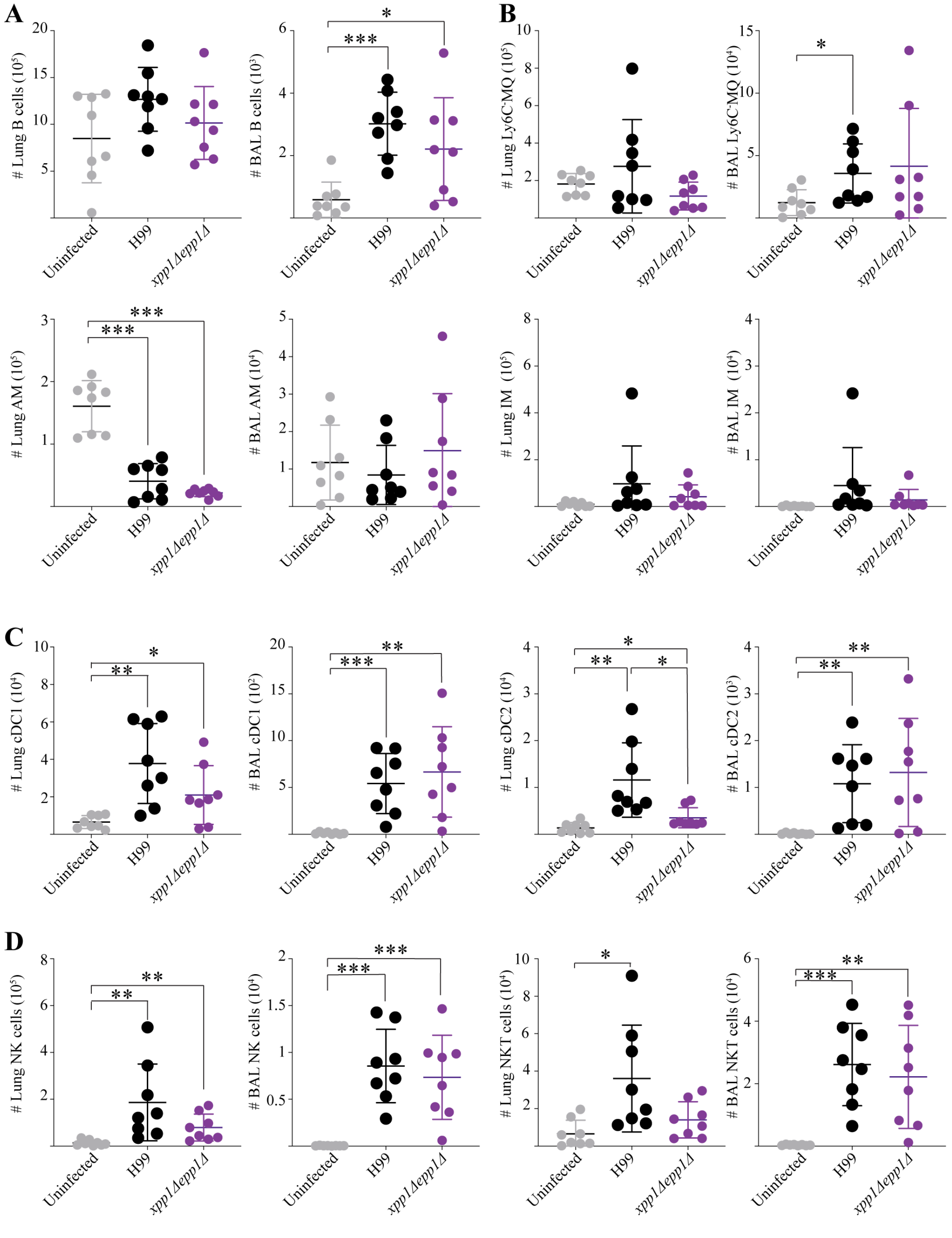

Figure S3. Infiltration of immune cells in the lung and BAL of mice infected with *C. neoformans*. Immune cell analysis in the BAL fluid or lung tissue of BALB/c mice infected with WT or *xpp1*Δ*epp1*Δ, or treated with physiological saline at 7 days post infection. Gating strategy was described previously in Figure S2. Total number of adaptive B cells (**A**), Ly6C^{-}macrophages, including alveolar macrophages (AM) and interstitial macrophages (IM) (**B**), and innate immune cells, including cDC1, cDC2, NK and NKT cells (**C**) in the lung and BAL. Data are presented as mean ± SD and representative of at least 2 independent experiments for each time point (n = 4 mice/time point). Significance indicated as *, P < 0.05; **, P < 0.01; ***, P < 0.001; unpaired Student t test.
