# Supplementary material for: Polyphosphatases have a polyphosphate-independent influence on the virulence of Cryptococcus neoformans
Source: Infect Immun. 2025 Mar 12;93(4):e00072-25. doi: 10.1128/iai.00072-25 (PMC11977306; doi:10.1128/iai.00072-25)
Supplement: Fig. S4 — The xpp1Δepp1Δ mutant provokes an altered immune response characterized by significantly reduced immune cell populations in lung tissue. [file iai.00072-25-s0004.pdf]

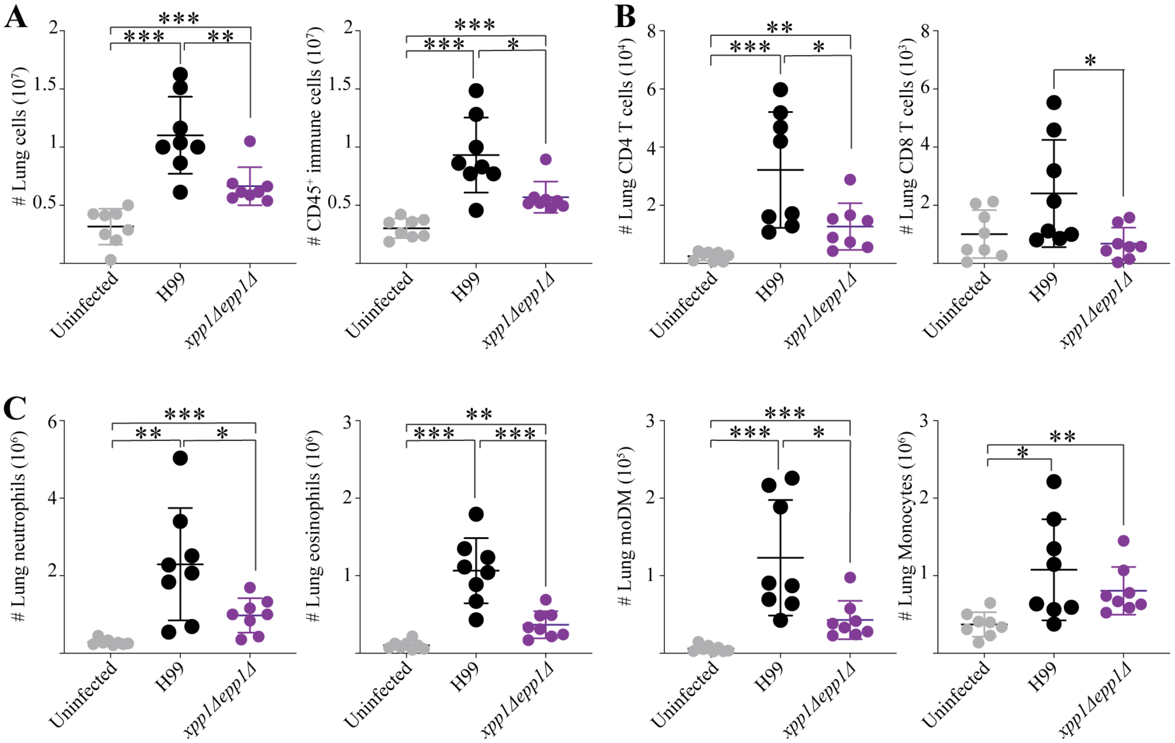

Figure S4. The *xpp1Δepp1Δ* mutant provokes an altered immune response characterized by significantly reduced immune cell populations in lung tissue. Immune cell analysis in the lung tissue of BALB/c mice infected with WT or *xpp1Δepp1Δ*, or treated with physiological saline at 7 dpi. **(A)** Total number of lung cells and $CD45^+$ leukocytes, **(B)** adaptive CD4 and CD8 T cells, and **(C)** innate immune cells, including neutrophils, eosinophils, moDM and monocytes in the lungs. Data are presented as mean ± SD and representative of at least 2 independent experiments for each time point (n = 4 mice/time point). Significance indicated as *, $P < 0.05$; **, $P < 0.01$; ***, $P < 0.001$; unpaired Student *t* test.
