# Supplementary material for: Polyphosphatases have a polyphosphate-independent influence on the virulence of Cryptococcus neoformans
Source: Infect Immun. 2025 Mar 12;93(4):e00072-25. doi: 10.1128/iai.00072-25 (PMC11977306; doi:10.1128/iai.00072-25)
Supplement: Fig. S5 — The vtc4Δepp1Δxpp1Δ triple deletion mutant does not have detectable polyP and exhibits sensitivity to zinc. [file iai.00072-25-s0005.pdf]

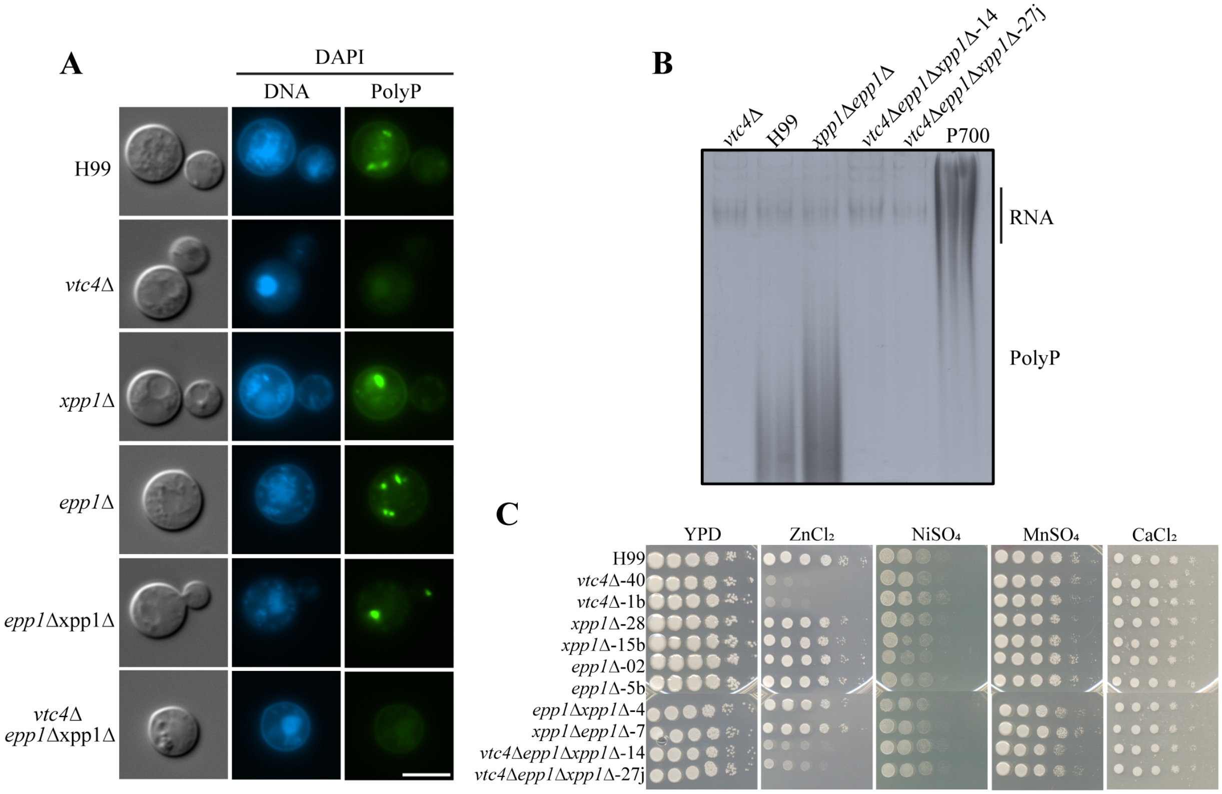

Figure S5. The *vtc4Δepp1Δxpp1Δ* triple deletion mutant does not have detectable polyP and exhibits sensitivity to zinc. (A) Representative widefield fluorescence micrographs showing polyP granules in WT, *xpp1Δ*, *epp1Δ*, *xpp1Δepp1Δ*, or *vtc4Δepp1Δxpp1Δ* (ΔΔΔ) strains. Indicated strains were stained with 4′,6-diamidino-2-phenylindole (DAPI, 100 µg/mL) for 30 minutes. Scale bar 5 µm. **(B)** Representative native polyacrylamide gel stained with toluidine blue O showing polyP accumulation in indicated strains. A marker of polyP type 700 (10 µg) was loaded alongside 10 µg of RNA extracted from each strain (cultured overnight in YPD). Data representative of at least three independent experiments. **(C)** Indicated strains were serially diluted and spotted onto solid YPD agar with or without 2.5 mM $ZnCl_2$, 3.0 mM $NiSO_4$, 1.875 mM $MnSO_4$, or 0.5 M $CaCl_2$. The plates were then incubated at 30 °C for 2-5 days before being photographed.
