# Supplementary material for: Polyphosphatases have a polyphosphate-independent influence on the virulence of Cryptococcus neoformans
Source: Infect Immun. 2025 Mar 12;93(4):e00072-25. doi: 10.1128/iai.00072-25 (PMC11977306; doi:10.1128/iai.00072-25)
Supplement: Fig. S6 — Loss of polyP synthesis and mobilization influences virulence in C57BL/6 mice. [file iai.00072-25-s0006.pdf]

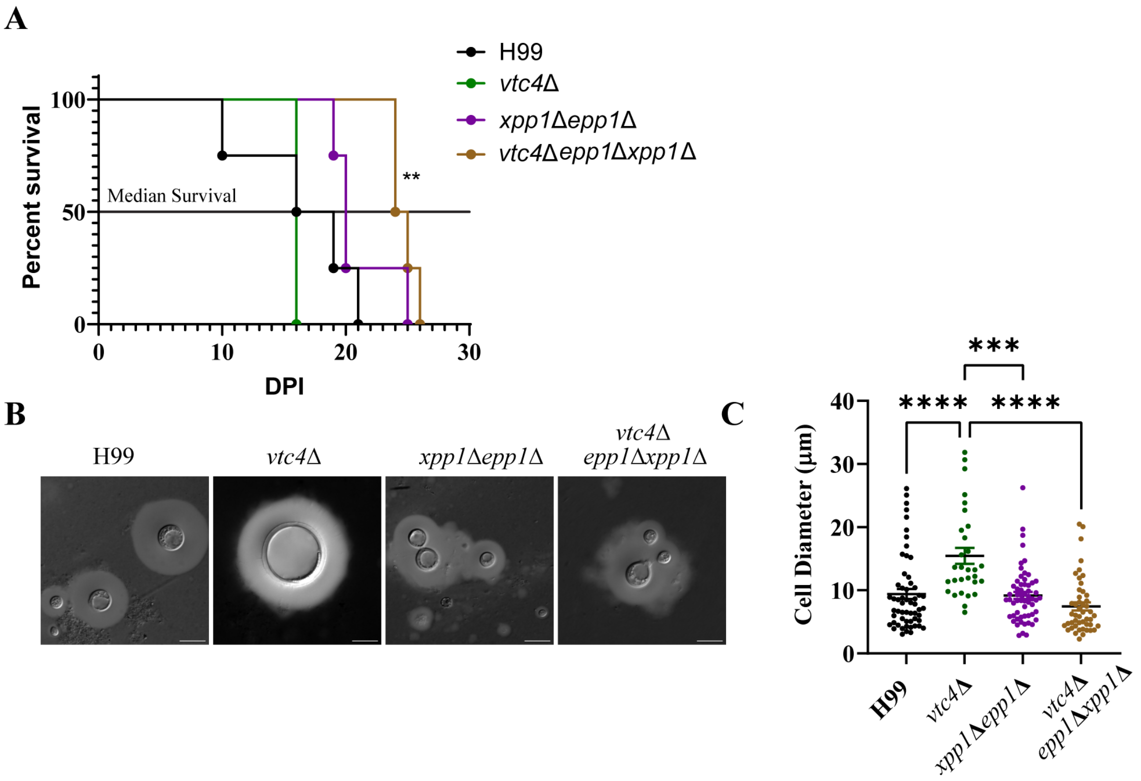

Figure S6. Loss of polyP synthesis and mobilization influences virulence in C57BL/6 mice. (A) Survival of C57BL/6 mice intranasally inoculated with cells of WT strain (H99), *vtc4*Δ mutant, *epp1*Δ*xpp1*Δ double mutant or *vtc4*Δ*epp1*Δ*xpp1*Δ triple mutant. The solid black horizontal line indicates median survival of mice. Yeast cells of indicated strains were isolated from the lungs of infected mice (n = 4) and **(B)** stained with India ink to visualize polysaccharide capsules. (**C**) Cell size was measured manually and at least 50 cells were analyzed per strain. Representative differential interference contrast (DIC) micrographs are shown. Scale bar 10 µm. Data are presented as mean ± SEM. Significance indicated as *, $P < 0.05$; **, $P < 0.01$; ***, $P < 0.001$; ****, $P < 0.0001$ one-way ANOVA or log-rank test.
