# Supplementary material for: Polyphosphatases have a polyphosphate-independent influence on the virulence of Cryptococcus neoformans
Source: Infect Immun. 2025 Mar 12;93(4):e00072-25. doi: 10.1128/iai.00072-25 (PMC11977306; doi:10.1128/iai.00072-25)
Supplement: Fig. S7 — Mutants deficient in polyP synthesis and mobilization are not sensitive to cell wall stress. [file iai.00072-25-s0007.pdf]

A

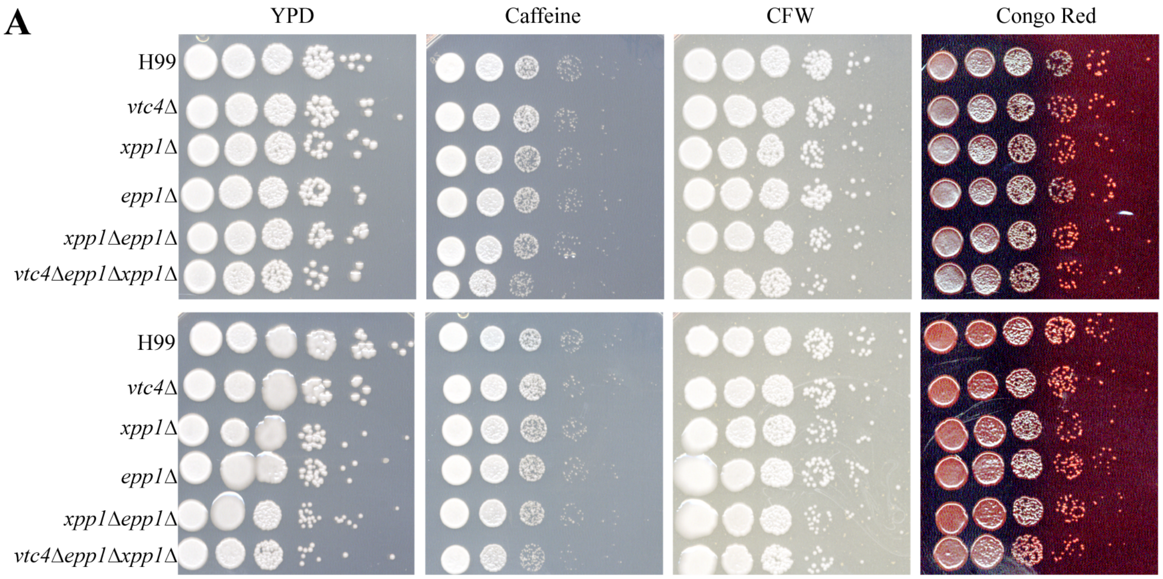

Figure S7. Mutants deficient in polyP synthesis and mobilization are not sensitive to cell wall stress. Indicated strains were serially diluted and spotted onto solid YPD agar with or without 1 mg/mL Caffeine, 1 mg/mL CFW or 1% Congo Red. The plates were then incubated at 30 °C for 5 days before being photographed.
