# Supplementary material for: Polyphosphatases have a polyphosphate-independent influence on the virulence of Cryptococcus neoformans
Source: Infect Immun. 2025 Mar 12;93(4):e00072-25. doi: 10.1128/iai.00072-25 (PMC11977306; doi:10.1128/iai.00072-25)
Supplement: Fig. S8 — Loss of polyP synthesis and mobilization does not confer sensitivity to all ROS or ETC stress agents. [file iai.00072-25-s0008.pdf]

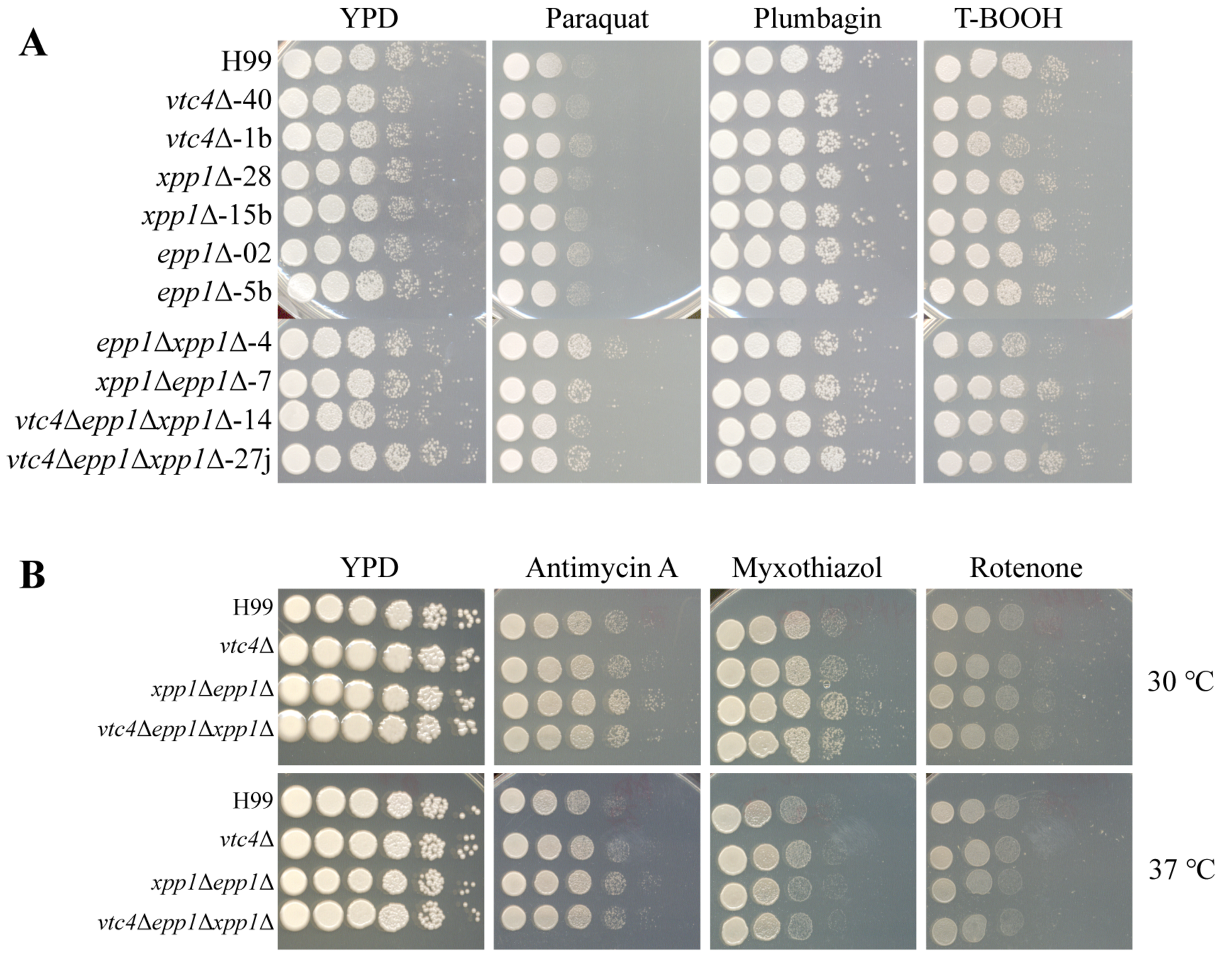

Figure S8. Loss of polyP synthesis and mobilization does not confer sensitivity to all ROS or ETC stress agents. Indicated strains were serially diluted and spotted onto solid YPD agar with or without 0.25 mM Paraquat dichloride, 25 µM Plumbagin, 1 mM tert-Butyl hydroperoxide (tBuOOH) **(A),** 5 µg/mL Antimycin A, 5 µM Myxothiazol, or 50 µg/mL Rotenone **(B)**. The plates were then incubated at 30°C or 37°C for 2-5 days before being photographed. **(C)** Liquid growth assays on YNB medium supplemented with 2% glucose, or 1% acetate for the indicated strains. *C. neoformans* yeast cells were grown in YNB without amino acids with 2% glucose or 1% acetate in test tubes containing 5 mL of the indicated growth medium. The tubes were incubated at 30°C, 200 rpm for 120 hours and optical densities (OD600) were measured every 24 hours. Data are presented as mean ± SEM representative of at least 3 independent experiments.
