# Supplementary material for: Polyphosphatases have a polyphosphate-independent influence on the virulence of Cryptococcus neoformans
Source: Infect Immun. 2025 Mar 12;93(4):e00072-25. doi: 10.1128/iai.00072-25 (PMC11977306; doi:10.1128/iai.00072-25)
Supplement: Supplemental tables — Tables S1 and S2. [file iai.00072-25-s0009.pdf]

S1 Table. *C. neoformans* strains used in this study.

Name	Description, Genotype	Source, Reference
H99/WT	*C. neoformans* wild type strain, serotype A	J. R. Perfect
*vtc4*Δ-40	*vtc4* deletion mutant in the H99 background, *vtc4*Δ*::NEO*	(5)
*vtc4*Δ-1b	*vtc4* deletion mutant in the H99 background, *vtc4*Δ*::NEO*	(5)
*xpp1*Δ-28	*xpp1* deletion mutant in the H99 background, *xpp1*Δ*::NAT*	(5)
*xpp1*Δ-15b	*xpp1* deletion mutant in the H99 background, *xpp1*Δ*::NAT*	(5)
*epp1*Δ-2	*epp1* deletion mutant in the H99 background, e*pp1*Δ*::NEO*	(5)
*epp1*Δ-5b	*epp1* deletion mutant in the H99 background, e*pp1*Δ*::NEO*	(5)
*epp1*Δ*xpp1*Δ-4	*epp1* deletion mutant in the *xpp1*Δ*-5 deletion mutant background*, *epp1*Δ*::NEO*	(5)
*xpp1*Δ*epp1*Δ-7	*xpp1* deletion mutant in the e*pp1*Δ*-2 deletion mutant background*, x*pp1*Δ*::NAT*	(5)
*vtc4*Δ*epp1*Δ*xpp1*-14	*vtc4* deletion mutant in the *epp1*Δ*xpp1*Δ-4 background, *vtc4*Δ*::HYG*	This study
*vtc4*Δ*epp1*Δ*xpp1*-27j	*vtc4* deletion mutant in the *epp1*Δ*xpp1*Δ-4 background, *vtc4*Δ*::HYG*	This study

S2 Table. Primers used for PCR amplification of the gene deletion constructs and for mutant screening.

Primers	Sequence (5' to 3')
ΔΔΔ-1	CGGCGACATGACGTGTATGCTC
ΔΔΔ-2	CGTAATCATGGTCATAGCTGTTTCCTGTATAGGGCAGGGACGGATGTGG
ΔΔΔ-3	CCACATCCGTCCCTGCCCTATACAGGAAACAGCTATGACCATGATTACG
ΔΔΔ-4	CATTACACTGGCTAACAACGCCCACGACGTTGTAAAACGACGGCCAG
ΔΔΔ-5	CTGGCCGTCGTTTTACAACGTCGTGGGCGTTGTTAGCCAGTGTAATG
ΔΔΔ-6	CAAGGAAGATTAGCGCCAAAGC
ΔΔΔ-7N	GGACCATGGAATTCAACGAC
ΔΔΔ-8N	TGCTCTTAGAAGGGGACAGG
HYGL	ACTCGCCGATAGTGGAAACCGA
HYGR	GCCACTCGAATCCTGCATGCT
